# CRISPR/Cas-mediated mRNA knockdown in the embryos of *Xenopus tropicalis*

**DOI:** 10.1186/s13578-025-01397-8

**Published:** 2025-04-23

**Authors:** Xiao-Lin Lin, Yi-Min Zhou, Ke Meng, Jia-Yi Yang, Han Zhang, Jin-Hua Lin, Hai-Yan Wu, Xiao-Yu Wang, Hui Zhao, Shan-Shan Feng, Kyu-Sang Park, Dong-Qing Cai, Li Zheng, Xu-Feng Qi

**Affiliations:** 1 Key Laboratory of Regenerative Medicine of Ministry of Education, Department of Developmental & Regenerative Biology, Jinan University, Guangzhou, 510632 China; 2 Department of Hematology, First Affiliated Hospital, Jinan University, Guangzhou, 510632 China; 3 Division of Histology & Embryology, School of Medicine, Jinan University, Guangzhou, 510632 China; 4 School of Biomedical Sciences, Faculty of Medicine, The Chinese University of Hong Kong, Hong Kong SAR, China; 5 Department of Physiology, Wonju College of Medicine, Yonsei University, Wonju, Gangwon, 220-701 Korea; 6 School of Environmental Science and Engineering, Guangdong University of Technology, Guangzhou, 510006 China; 7 Key Laboratory of Regenerative Medicine of Ministry of Education, Jinan University, Guangzhou, 510632 China

**Keywords:** Cas13, CRISPRi, Knockdown, *Xenopus tropicalis*

## Abstract

The *Xenopus tropicalis* (Western clawed frog) is an important amphibian model for genetics, developmental and regenerative biology, due to its diploid genetic background and short generation time. CRISPR-Cas13 and CRISPR interference (CRISPRi) systems have recently been employed to suppress mRNA expression in many organisms such as yeast, plants, and mammalian cells. However, no systematic study of these two systems has been carried out in *Xenopus tropicalis*. Here, we show that CRISPRi rather than CRISPR-Cas13 is an effective and suitable approach to suppress specific mRNA transcription in *Xenopus tropicalis* embryos. We demonstrated that CRISPRi composed of dCas9 and KRAB-MeCP2 (dCas9-KM) can efficiently target exogenous and endogenous transcripts in *Xenopus tropicalis* embryos. Moreover, our data suggest that the new KRAB domain from ZIM3 protein (ZIM3-KRAB, ZIM3K) alone has a comparable transcript targeting capacity in *Xenopus tropicalis* embryos to the traditional fusion repressor KRAB-MeCP2 in which the KRAB domain from KOX1 protein. In conclusion, our results demonstrate that CRISPRi rather than CRISPR-Cas13 is an efficient knockdown platform to explore specific gene function in *Xenopus tropicalis* embryos.

**Supplementary Information:**

The online version contains supplementary material available at 10.1186/s13578-025-01397-8.

## Introduction

The *Xenopus tropicalis* (Western clawed frog) is an important amphibian model for genetic and embryonic studies, due to its true diploid genetic background and short generation time 1, 2. Gene knockout in *X. tropicalis* has been well developed using genomic editing technologies including the type II clustered regularly interspaced short palindromic repeats (CRISPR)/CRISPR-associated (Cas) 9 system (CRISPR/Cas9) 3–5. This strategy allows researchers to link genotype to phenotype in *Xenopus* through the generation of permanent changes in the genome. However, gene knockdown approaches such as RNA interference (RNAi) have largely failed in *Xenopus*, although RNAi is an efficient tool to suppress target gene expression in many other organisms 6. Morpholinos (MOs), the nucleic acid-analog antisense oligomers, have been used for over two decades in *Xenopus* to disrupt gene function by blocking translation or splicing 7. However, recent studies have reported that MOs utility induced toxicity and off-target effects, which calling MOs into question 8–10. Compared with gene knockout based on DNA mutagenesis, RNA knockdown approaches hold many advantages. Knockout strategy needs a multi-generation genotype screening to establish a permanent genetic strain. However, knockdown approach can directly disrupt gene activity and allow researchers to study gene function as soon as in F0 funders. Moreover, researchers can study how subtle changes in transcript levels impact biological processes by using knockdown rather than knockout approaches. Importantly, the function of some embryonic-essential genes can only be uncovered by using knockdown approach, but not via knockout strategy due to their loss-of-function lethality 11. In addition, targeting RNA rather than DNA is more safe, because RNA targeting is temporary and effective modification without the impact of permanent heritable changes 12.

Cas13 proteins are class 2 type VI clustered regularly interspaced short palindromic repeats (CRISPR) effectors, which allow for target gene knockdown without altering the genome. Cas13 proteins are guided to target RNAs by a guide RNA (gRNA) through RNA-RNA hybridization. Cas13 system has recently been employed to induce both the cleavage and subsequent degradation of targeting RNAs in mammalian cells 12–15, zebrafish 16, and plants 17. It has been reported that Cas13 system is more effective and specific than RNAi to silence target mRNAs in mammalian cells 12, 14, 18. It has been reported that the effective Cas13 system contains several popular subtypes including LwaCas13a, PspCas13b, RfxCas13d. Compared with LwaCas13a with knockdown efficiency of 40.1%, PspCas13b could reach an average knockdown efficiency of 92.3%, indicating that PspCas13b may have greater potential in scientific and technological applications 19. On the other hand, Cas13d is the smallest effector protein in the type VI CRISPR/Cas system, therefore it could be more conducive to the construction and packaging of expression vectors 13, 20.To date, PspCas13b and RfxCas13d have been reported as showing high RNA knockdown efficacy with minimal off-target activity 12, 14, 15.

CRISPR interference (CRISPRi), based on the catalytically dead Cas9 (dCas9) and a gRNA to generate a DNA recognition complex that interfere with transcription, is a powerful platform for silencing gene expression 21. Kruppel-associated boxs (KRAB) are potent transcriptional repression domains. It was first functionally characterized from KOX1 (ZNF10) protein 22. The initial CRISPRi system was further developed by fusing dCas9 with the KRAB domain of KOX1, known as dCas9-KRAB (dCas9-K), to improve the efficacy of CRISPRi 23. In the past several years, numerous studies had demonstrated that dCas9-K system can effectively silence target gene expression in mammalian cells 23–25. Methyl-CpG binding protein 2 (MeCP2) is a multifunctional epigenetic reader playing a role in transcriptional regulation and chromatin structure 26. Recently, a systematic study has developed a bipartite construct of MeCP2 fused to dCas9-KRAB (known as dCas9-KRAB-MeCP2, dCas9-KM), which can greatly enhance the repressive activity of dCas9-K system in mammalian cells 27.

Although Cas13 and CRISPRi systems have been proposed to be efficient for RNA targeting in many organisms, their potential roles in *X. tropicalis* remain unclear. To date, no systematic study of these two systems has been carried out in *X. tropicalis*. In the present study, we showed that CRISPRi rather than Cas13 is an effective and suitable approach to suppress specific mRNA transcripts in *X. tropicalis* embryos.

## Results

### Effects of Cas13 systems on reporter gene expression in embryos of *X. tropicalis*

To date, PspCas13b and RfxCas13d are the two Cas13 effector proteins with high RNA knockdown efficacy in mammalian cells 12, 14. To confirm their RNA knockdown efficacy in mammalian cells, plasmids expressing PspCas13b and RfxCas13 were transfected into 293T cells together with individual corresponding guide RNA (gRNA) expressing plasmids. EGFP was used as a reporter gene in this study. Three gRNAs targeting EGFP (gEGFP1-3) were designed for these two Cas13 effectors, respectively (Fig. S1A-C). The gRNA targeting LacZ was used as negative control (gNC) 12. After transfection for 48 h, EGFP expression was evaluated by fluorescence microscopy and Flow cytometry, respectively. Our data showed that each gEGFP greatly decreased EGFP expression by about 50% in PspCas13b system (Fig. S1D and E). However, more than 80% EGFP expression was reduced by each gEGFP in RfxCas13d system (Fig. S1F and G). These findings confirmed the robust RNA knockdown efficacy of PspCas13b and RfxCas13d systems in mammalian cells as previous reported 12, 14.

**Fig. 1:**
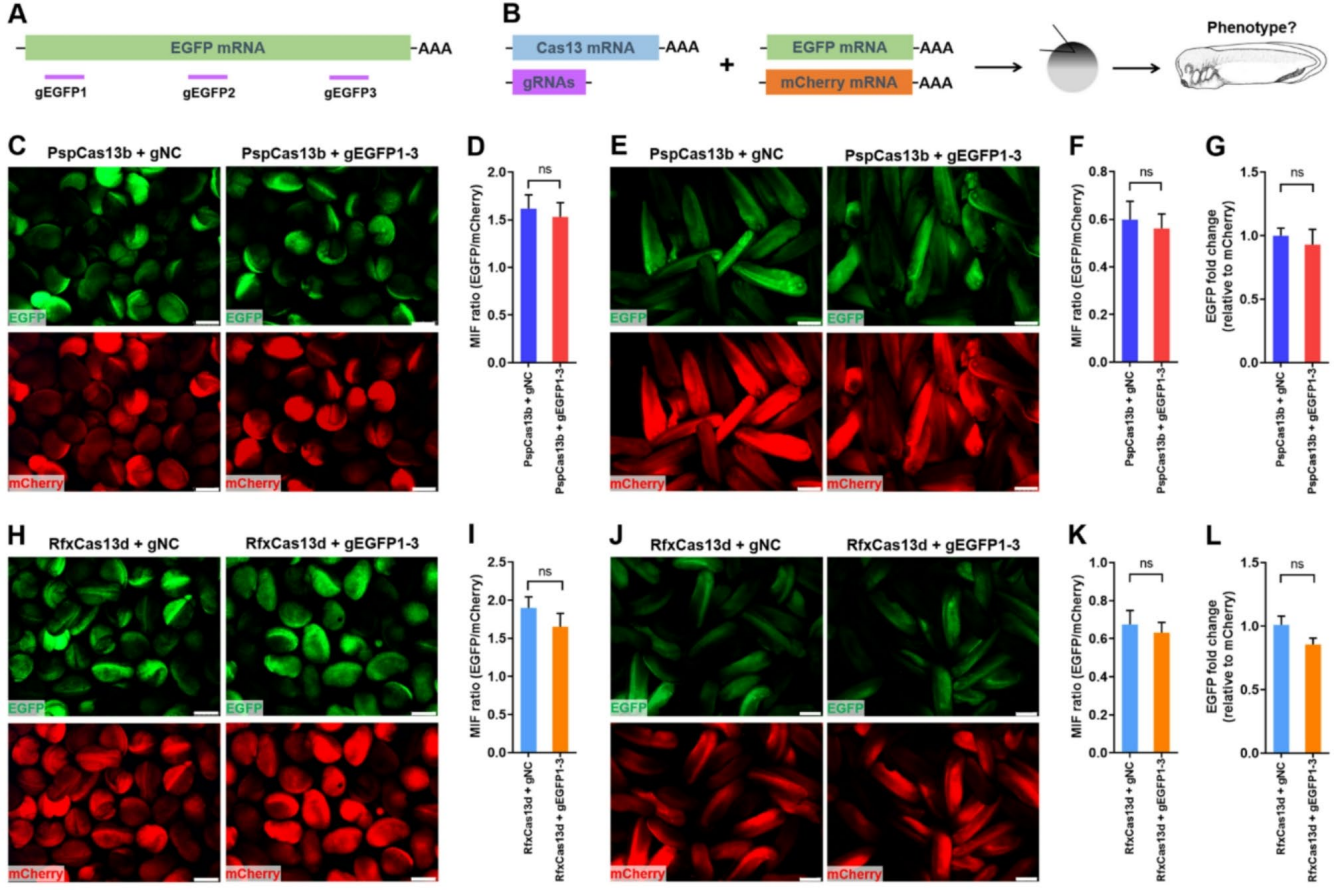
Effects of PspCas13b and RfxCas13d on reporter gene expression in embryos of *X. tropicalis*. (**A**) Schematic of Cas13-related gRNAs targeting the mRNA of EGFP reporter gene. (**B**) Schematic illustration of the experimental setup used to analyze PspCas13b and RfxCas13d capacities to target exogenous reporter gene in *X. tropicalis* embryos. The mRNA of mCherry was used as the internal control. (**C** and **D**) Representative images (**C**) and quantification (**C**) of EGFP fluorescence in control and experimental embryos injected with PspCas13b system at 15 hpi (*n* = ~ 30 embryos from 3 independent experiments). (**E** and **F**) Representative images (**E**) and quantification (**F**) of EGFP fluorescence in control and experimental embryos injected with PspCas13b system at 24 hpi (*n* = ~ 30 embryos from 3 independent experiments). (**G**) The qPCR validation of EGFP expression in control and experimental embryos injected with PspCas13b system at 24 dpi (*n* = 5). (**H** and **I**) Representative images (**H**) and quantification (**I**) of EGFP fluorescence in control and experimental embryos injected with RfxCas13d system at 15 hpi (*n* = ~ 30 embryos from 3 independent experiments). (**J** and **K**) Representative images (**J**) and quantification (**K**) of EGFP fluorescence in control and experimental embryos injected with RfxCas13d system at 24 hpi (*n* = ~ 30 embryos from 3 independent experiments). (**L**) The qPCR validation of EGFP expression in control and experimental embryos injected with RfxCas13d system at 24 dpi (*n* = 5). All data are presented as mean ± SEM. Scale bar = 500 μm (**C**, **E**, and **J**) and 750 μm (**H**). Ns, no significant differences

Subsequently, we examined the potential efficacy of Cas13 systems on reporter gene expression in *X. tropicalis*. Cas13 mRNA, gRNA mix (gEGFP1-3), EGFP mRNA (target), and mCherry mRNA (reference gene) were co-injected into the animal pole of *X. tropicalis* fertilized eggs at one-cell stage, followed by fluorescence evaluation at 15 and 24 h post-injection (hpi), respectively (Fig. 1A and B). Our data showed that the co-injection of PspCas13b and gEGFP1-3 could not significantly decrease EGFP expression in embryos of *X. tropicalis* compared with gNC groups, which indicating that PspCas13b system failed to reduce the expression of reporter gene in *X. tropicalis* (Fig. 1C-G). For RfxCas13d system, there were no significant differences in EGFP expression between gEGFP1-3 and control groups, implying that RfxCas13d system does not influence reporter gene expression in embryos of *X. tropicalis* (Fig. 1H-L). Next, we examined the effects of other Cas13d orthologs (AdmCas13d, EsCas13d, p1E0Cas13d, and RffCas13d) 14 on the expression of reporter gene in *X. tropicalis*. In agreement with RfxCas13d, our data showed that reporter gene expression was not disrupted by these four Cas13d orthologs (Fig. S2).

### Effects of Cas13 systems on endogenous gene expression in embryos of *X. tropicalis*

**Fig. 2:**
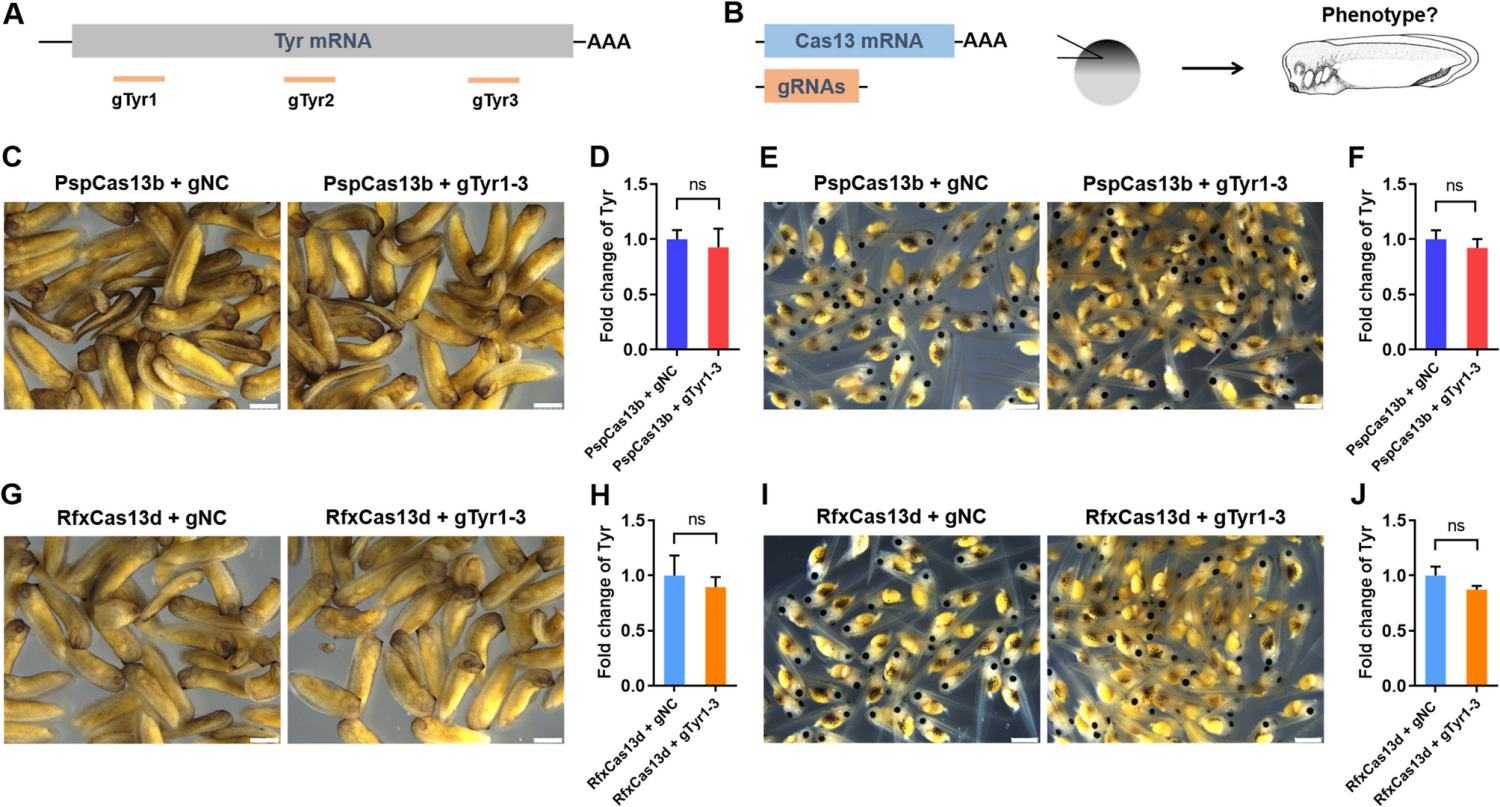
Effects of PspCas13b and RfxCas13d on tyrosinase expression in embryos of *X. tropicalis*. (**A**) Schematic of Cas13-related gRNAs targeting the mRNA of endogenous tyrosinase (*tyr*) gene. (**B**) Schematic illustration of the experimental setup used to analyze the capacities of PspCas13b and RfxCas13d systems (300 pg/embryo) together with gTyr1-3 (100 pg/embryo). (C and D) Representative images (**C**) and quantification (**D**) of Tyr expression in control and experimental embryos injected with PspCas13b system at 24 hpi. Scale bar = 500 μm. (**E** and **F**) Representative images (**E**) and quantification (**F**) of Tyr expression in control and experimental embryos injected with PspCas13b system at 48 hpi. Scale bar = 1 mm. (**G** and **H**) Representative images (**G**) and quantification (**H**) of Tyr expression in control and experimental embryos injected with RfxCas13d system at 24 hpi. Scale bar = 500 μm. (**I** and **J**) Representative images (**I**) and quantification (**J**) of Tyr expression in control and experimental embryos injected with RfxCas13d system at 48 hpi. Scale bar = 1 mm. All data are presented as mean ± SEM (*n* = 4 per group). Ns, no significant differences

To further explore whether Cas13 systems can influence the expression of endogenous gene in *X. tropicalis*, we chose tyrosinase (*tyr*) as the endogenous target gene due to its disruption causes the ablation of pigmentation 4. We designed three gRNAs targeting the *tyr* (gTyr1-3) in *X. tropicalis* (Fig. 2A). Cas13 mRNA (300 pg/embryo) and gRNA mix (gTyr1-3, 100 pg/embryo) were co-injected into the animal pole of *X. tropicalis* fertilized eggs at one-cell stage, followed by evaluation of *tyr* expression at 24 and 48 hpi, respectively (Fig. 2B). For PspCas13b system, microscopy images showed that there were no significant differences in the pigmentation between gTyr1-3 and control groups at 24 hpi (Fig. 2C). Consistently, quantitative real-time PCR (qPCR) revealed that *tyr* mRNA expression was not significantly suppressed by gTyr1-3 compared with gNC at 24 hpi (Fig. 2D). To test whether extending the duration of PspCas13b system can induce target gene ablation in *X. tropicalis*, we further evaluated *tyr* expression at 48 hpi. In agreement with 24 hpi, there was no significant differences in pigmentation and *tyr* expression levels between gTry1-3 and gNC groups at 48 hpi (Fig. 2E and F). These data indicate that PspCas13b system cannot significantly inhibit the expression of endogenous gene in *X. tropicalis* embryos. In agreement with PspCas13b, RfxCas13d system did not induce significant disruption of pigmentation and *tyr* expression levels in embryos of *X. tropicalis* at 24 and 48 hpi compared with control groups (Fig. 2G-J).

Subsequently, high dose of gTyr1-3 (600 pg/embryo) was used to determine the capacity of Cas13 systems. In consistent with low dose of gTyr1-3, our data showed that co-injection of PspCas13b mRNA and gTyr1-3 did not significantly affect the expression of *tyr* at 24 and 48 hpi (Fig. S3A and B). In line with PspCas13b, no significant differences in *tyr* expression levels were detected in embryos co-injected with RfxCas13d mRNA and gTyr1-3 when compared with gNC group (Fig. S3C and D). Except for PspCas13b and RfxCas13d, LwaCas13a is another popular Cas13 protein that can disrupt target gene expression in mammalian and plant cells 18. To test whether high dose of gTyr1-3 disrupt the endogenous gene expression in *X. tropicalis*, we co-injected LwaCas13a mRNA and gTyr1-3 into embryos and found that LwaCas13a system had no significant effects on the expression of tyr at 48 hpi (Fig. S4). These data reveal that increasing the concentration of gRNAs for Cas13 systems cannot effectively suppress the expression of endogenous target in *X. tropicalis*. Among these three Cas13 systems, RfxCas13d has been proved to be the strongest effector to knock down the expression of the targets in mammalian cells 14, 15. To further determine the potential of RfxCas13d in *X. tropicalis*, we designed another three gRNAs targeting *tyr* gene (gTyr4-6 set including gTyr4, gTyr5, and gTyr6). In agreement with the results using gTyr1-3, no significant differences in *tyr* expression were detected in embryos co-injected with RfxCas13d mRNA and gTyr4-6 (600 pg/embryo) when compared with gNC group (Fig. S5). Taken together, these findings suggest that Cas13 system might be not suitable for RNA knockdown in the embryos of *X. tropicalis*.

These observation prompt us to ask whether these Cas13 proteins are normally expressed in in *X. tropicalis* or not. To confirm this issue, we constructed plasmids expressing the Cas13-EGFP fusion protein (pCAG-PspCas13b-EGFP and pCAG-RfxCas13d-EGFP). The plasmid pCAG-EGFP was used as control (Fig. S6A). These plasmids were transfected into 293T cells for 48 h. In agreement with EGFP alone, our data showed that PspCas13b-EGFP and RfxCas13d-EGFP fusion proteins could be expressed in mammalian cells as proved by the production of EGFP reporter (green fluorescence) (Fig. S6B). Subsequently, we injected these plasmids into embryos (600 pg/embryo) to determine reporter gene expression. Our data showed that the EGFP reporter production was detected in embryos injected with pCAG-EGFP. In contrast, there were no visible fluorescent signals in embryos injected with pCAG-PspCas13b-EGFP and pCAG-RfxCas13d-EGFP plasmids (Fig. S6C and D). To further exclude the potential influence of CAG promoters, we also injected the in vitro transcribed mRNAs including EGFP, PspCas13b-EGFP and RfxCas13d-EGFP, followed by the determination of reporter gene expression at 24 ~ 72 h. We found that EGFP reporter production was detected in embryos injected with EGFP mRNA. However, almost no fluorescent signals were detected in embryos injected with PspCas13b-EGFP and RfxCas13d-EGFP mRNAs (Fig. S7). These data suggest that Cas13 system may not be normally expressed in *X. tropicalis*.

### Effects of CRISPRi on reporter gene expression in embryos of *X. tropicalis*

**Fig. 3:**
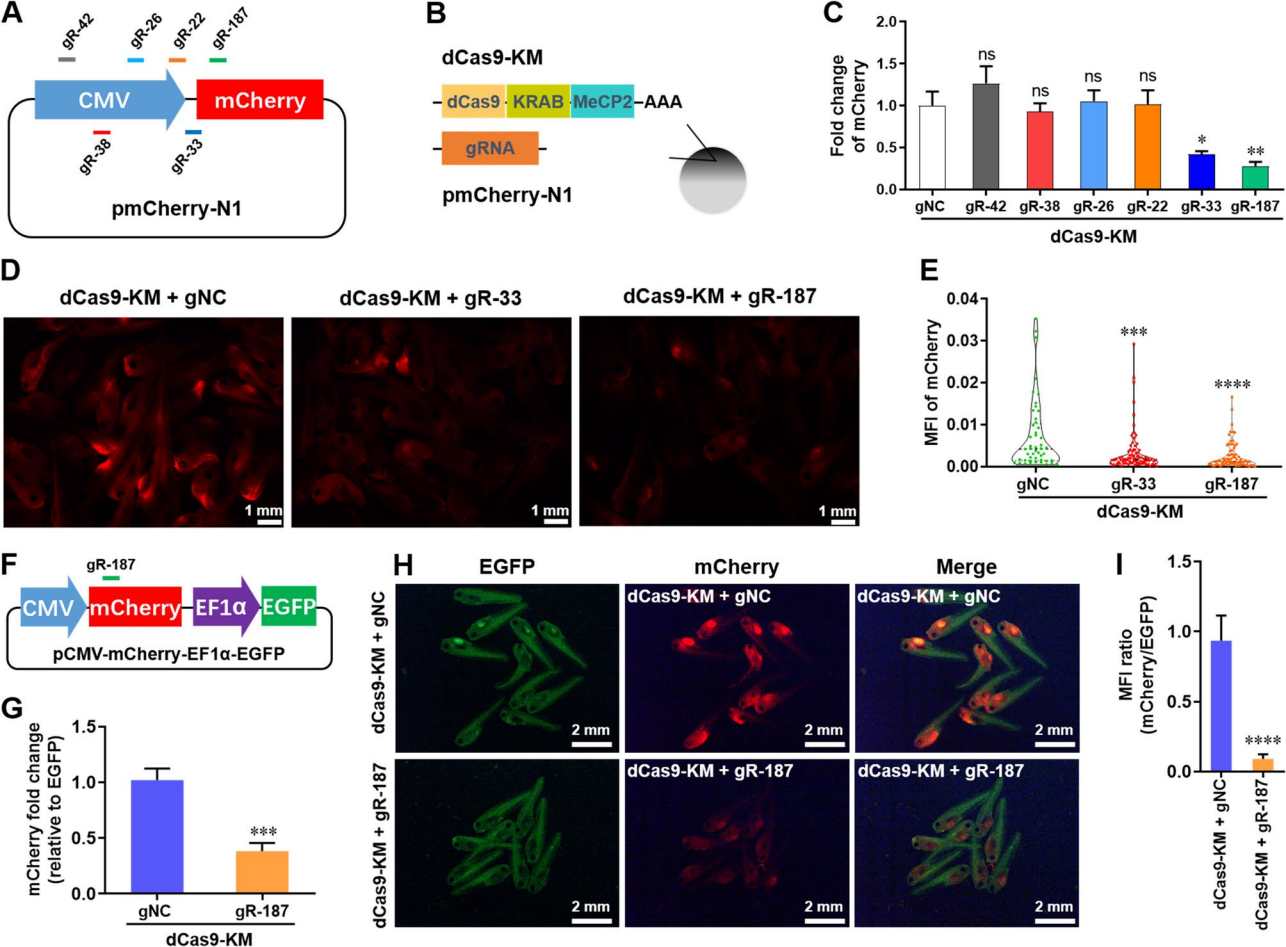
Effects of dCas9-KM system on reporter gene expression in embryos of *X. tropicalis*. (**A**) Schematic of CRISPRi-related gRNAs targeting the regions of mCherry reporter gene in pmCherry-N1 plasmid. (**B**) Schematic illustration of the experimental setup used to analyze dCas9-KM capacity targeting exogenous reporter gene in *X. tropicalis* embryos. (**C**) The qPCR validation of mCherry expression in embryos co-injected with dCas9-KM system and indicated gRNAs at 48 dpi (*n* = 4 per group). (**D** and **E**) Representative images (**D**) and quantification (**E**) of mCherry expression in embryos co-injected with dCas9-KM system and indicated gRNA at 48 hpi (*n* = ~ 60 embryos from 3 independent experiments). (**F**) Schematic of gR-187 targeting mCherry reporter gene in pCMV-mCherry-EF1α-EGFP dual-reporter plasmid. (**G**) The qPCR validation of mCherry expression in embryos co-injected with dCas9-KM system and indicated gRNA at 48 dpi (*n* = 5 per group). EGFP was used as the internal control. (**H** and **I**) Representative images (**H**) and quantification (**I**) of mCherry fluorescence in embryos co-injected with dCas9-KM system and indicate gRNA at 48 hpi (*n* = 24 embryos from 3 independent experiments). All data are presented as mean ± SEM. *p < 0.05, **p < 0.01, ***p < 0.001, ****p < 0.0001 versus control. Ns, no significant differences versus control

CRISPRi is a platform for silencing gene expression based on inactive Cas9 (dCas9) alone or fusion with some repressors. To date, the fusion repressor dCas9-KRAB-MeCP2 (dCas9-KM) has been demonstrated to be the most powerful CRISPRi system for mammalian gene silencing 27. CRISPRi-mediated RNA knockdown has been reported in a variety of organisms. However, the potential role of CRISPRi system in *X. tropicalis* remains unclear. To evaluate the RNA knockdown activity of CRISPRi system in *X. tropicalis*, we established a reporter plasmid expressing mCherry driven by CMV promoter, and designed 6 guide RNAs (gRNAs) in the fragment of CMV promoter and 5’ end of target gene (Fig. 3A). The dCas9-KM mRNA and gRNA were co-injected into embryos together with reporter plasmid (Fig. 3B). Among these tested 6 gRNAs, qPCR assay revealed that the mRNA levels of mCherry were significantly inhibited by gR-33 and gR-187 rather than other four gRNAs (Fig. 3C). Consistently, decreased protein levels of mCherry were also detected in gR-33- and gR-187-injected groups (Fig. 3D and E). The gR-187 was chosen for further study due to its higher efficacy compared with gR-33. To further precisely confirmed gR-187-induced silencing of mCherry in *X. tropicalis*, we developed a dual-reporter system expressing mCherry and EGFP under different promoters on the same vector, which allowing the mCherry transcript to serve as the CRISPRi target and the EGFP to serve as a dosing control (Fig. 3F). Dual-reporter plasmid, dCas9-KM mRNA, and gR-187 were co-injected into the animal pole of fertilized eggs to examine its efficacy. Our data showed that gR-187 greatly decreased the expression of mCherry at 48 hpi compared with control, as demonstrated by the significant decreases in mRNA and protein levels (Fig. 3G). Taken together, these findings revealed that dCas9-KM-mediated CRISPRi system can greatly inhibit the expression of reporter gene in *X. tropicalis*.

To compare the efficacy of different types of CRISPRi systems in *X. tropicalis*, dCas9 with or without repressor domains were evaluated in embryos injected with gR-187 and dual-reporter plasmid (Fig. S8A). Our data showed that co-injection of dCas9 and gR-187 significantly reduced the mRNA levels of mCherry by about 37% at 24 hpi when compared with dCas9 alone (control). Moreover, mRNA levels of mCherry were greatly suppressed by more than 60% in embryos injected with dCas9-K and dCas9-KM systems compared with control group, respectively (Fig. S8B). We extended the incubation time of embryos to 48 hpi and found that these three types of CRISPRi systems further suppressed mCherry expression compared with 24 hpi, respectively. Among them, dCas9-KM system showed the most efficacy for targeting reporter gene expression (Fig. S8C). In consistent with mRNA levels, fluorescence images showed that three CRISPRi systems individually reduced the production of mCherry instead of EGFP, compared with control group (Fig. S8D). Quantification of mean fluorescence intensity (MFI) further confirmed that dCas9-KM system has the most efficacy for targeting mCherry production in *X. tropicalis*, as demonstrated by the most decrease in MFI ratio (mCherry/EGFP) (Fig. S8E). Thus, dCas9-KM system was chosen for further experiments in the following study.

### Effects of dCas9-KM on *tyr* expression in embryos of *X. tropicalis*

**Fig. 4:**
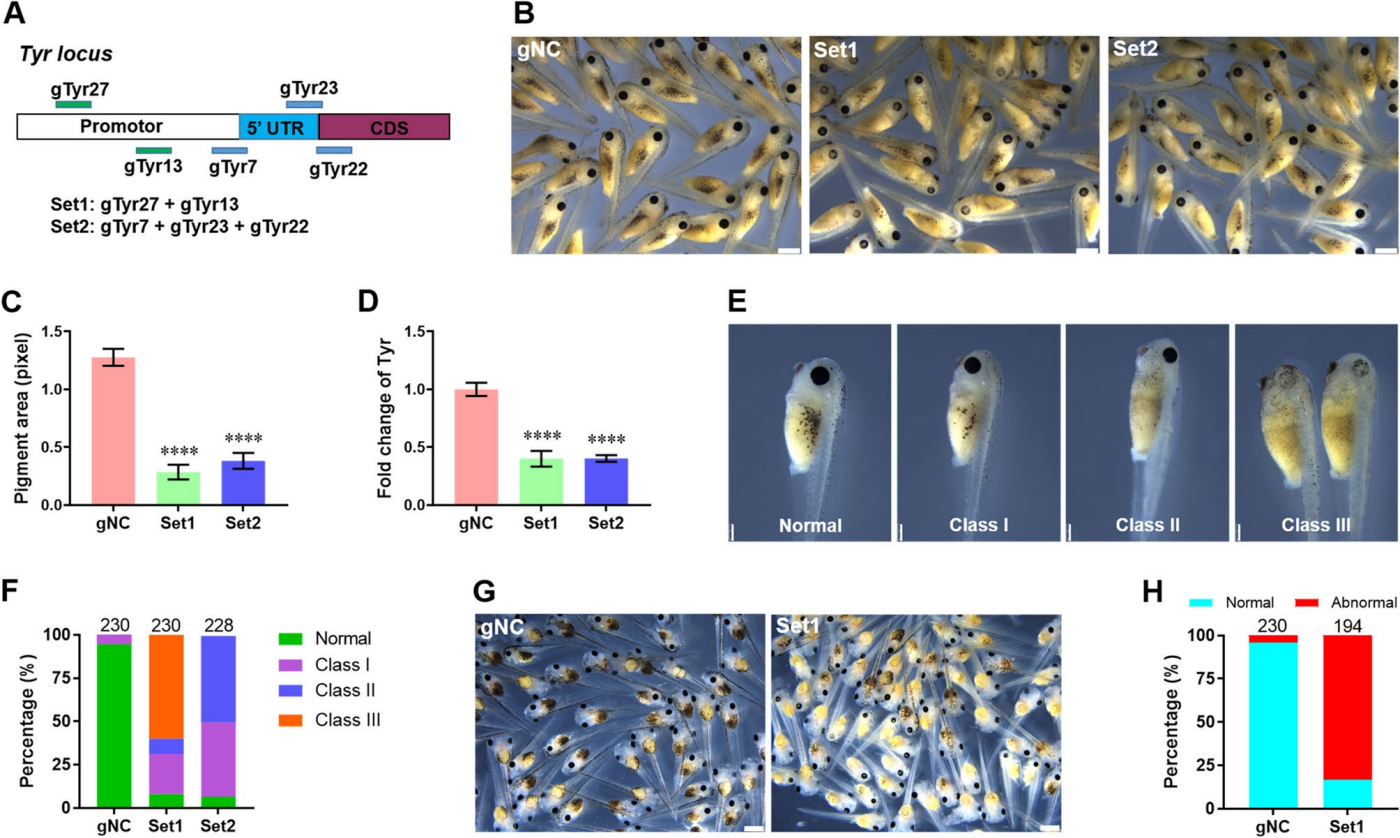
Effects of dCas9-KM system on tyrosinase expression in embryos of *X. tropicalis.* (**A**) Schematic illustration of dCas9-KM system-related gRNAs targeting the DNA locus of endogenous tyrosinase (Tyr) gene. Fertilized eggs were co-injected with dCas9-KM mRNAs and indicated gRNA sets, followed by embryo evaluation at 48 hpi as follows. (**B** and **C**) Representative images (**B**) and quantification (**C**) of Tyr production in embryos co-injected with dCas9-KM mRNA and indicated gRNAs at 48 hpi (*n* = 26 ~ 30 embryos from 3 independent experiments). Scale bar = 250 μm. (**D**) The qPCR validation of Tyr expression in embryos co-injected with dCas9-KM mRNA and indicated gRNAs at 48 dpi (*n* = 5 per group). (**E** and **F**) Representative images (**E**, scale bar = 250 μm) and quantification (**F**) of embryos with different phenotypes were counted and compared with the total developed ones 48 h post co-injection of dCas9-KM system. (**G** and **H**) Representative images (**G**) and quantification (**H**) of embryos with different phenotypes were counted and compared with the total developed ones 72 h post co-injection of dCas9-KM system. Total embryos evaluated for each group (n) is shown above each column. All data are present as mean ± SEM. ****p < 0.0001 versus gNC group

Subsequently, we further evaluated the potential roles of dCas9-KM system in targeting endogenous genes in *X. tropicalis*. We firstly chose *tyr* as the endogenous target and designed five gRNAs targeting the fragments of promoter, 5’ UTR, and 5’ end of CDS (Fig. 4A). These five gRNAs were divided into two sets including Set1 (gTyr27 + gTyr13) and Set2 (gTyr7 + gTyr23 + gTyr22) (Fig. 4A). The dCas9-KM mRNA and gRNAs were co-injected into the animal pole of *X. tropicalis* fertilized eggs at one-cell stage, followed by *tyr* expression evaluation at 48 hpi. Our data showed that both gRNA Sets greatly induced the ablation of pigmentation compared with control group (Fig. 4B and C). In addition to the partial albinism in body, many embryos with severe perturbation of pigmentation in eyes were observed in Set1-injected embryos compared with control (Fig. 4B). The qPCR assay further confirmed that the mRNA expression of *tyr* was significantly suppressed by gRNA Sets compared with control (Fig. 4D). In this work, ablation of pigmentation in *X. tropicalis* embryos was divided three classes. Class-I is a mild albinism phenotype denoting the tadpoles with little pigments in body. Class-II is a moderate albinism phenotype denoting the tadpoles almost without pigments in body. Class-III is the severe albinism phenotype denoting the tadpoles almost without pigments in body and eyes. Although more than 90% tadpoles had albinism phenotypes (ClassI-III) in Set1 and Set2 groups compared with gNC, most (about 60%) albinism tadpoles in Class III (severe albinism in body and eye) were induced by Set1 injection (Fig. 4E and F). We further incubated these tadpoles to 72 hpi and found that Set1 injection resulted in an approximately 83% of tadpoles with abnormal pigmentation (Fig. 4G and H).

To further determine which one in Set1 group possesses the capacity to inhibit *tyr* expression, individual gTyr was injected into embryos together with dCas9-KM. Pigmentation images and qPCR assay showed that gTyr13 rather than gTyr27 greatly suppressed pigmentation and *tyr* mRNA expression at 48 hpi when compared with gNC (Fig. S9A and B). In consistent with these data, phenotype quantification showed that gTyr13 injection greatly increased the percentage of tadpoles with abnormal pigmentation (albinism phenotype) compared with control group (Fig. S9C). To determine the effective duration of dCas9-KM system in *X. tropicalis*, abnormal tadpoles with albinism were identified and isolated from dCas9-KM/gTyr13-injected groups at 48 hpi (Fig. S10A). These isolated abnormal tadpoles were further incubated for a long time. We found that the albinism phenotype can be maintained for several days. The albinism phenotype was not reversed until 120 hpi as demonstrated by the slight increase in pigmentation at 120 hpi (Fig. S10B). To determine whether pigmentation loss can restore to normal levels with tadpole development, we raised these tadpoles to 9 days post-injection (dpi). We found that there were no obvious differences in the pigmentation of tadpoles between dCas9-KM/gTyr13- and dCas9-KM/gNC-injected groups (Figure S10C), indicating that dCas9-KM/gTyr13-induced pigmentation loss restores to normal levels at 9 dpi. These findings indicated that dCas9-KM system is an efficient and transient tool to trigger tyrosinase mRNA knockdown during *X. tropicalis* embryogenesis.

### Effects of dCas9-KM on other endogenous targets in embryos of *X. tropicalis*

**Fig. 5:**
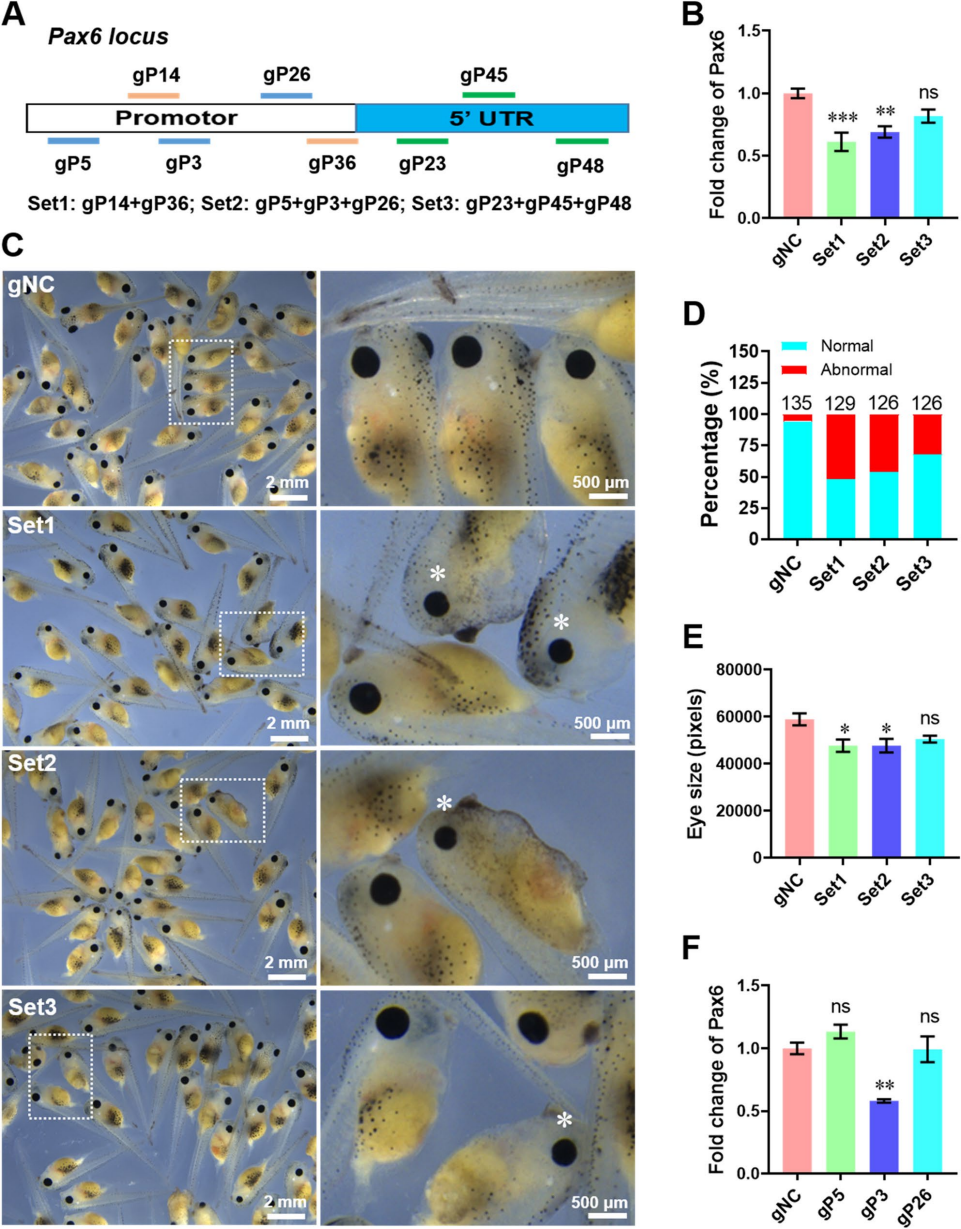
Effects of dCas9-KM system on pax6 expression in embryos of *X. tropicalis*. (**A**) Schematic illustration of dCas9-KM system-related gRNAs targeting the DNA locus of endogenous pax6 gene. Fertilized eggs were co-injected with dCas9-KM mRNAs and indicated gRNA sets, followed by embryo evaluation at 48 hpi as follows. (**B**) The qPCR validation of pax6 expression in the dCas9-KM-injected embryos with different gRNA sets (*n* = 5 per group). (**C**) Representative images of embryos with smaller eyes in the experimental group injected with pax6 targeting gRNA sets. The top panel shows low magnification, and the lower panel shows high magnification. Asterisk denotes smaller eyes. (**D**) Embryos with different phenotypes were counted and compared with the total developed ones after injection. Total embryos evaluated for each group (n) is shown above each column. (**E**) Quantification of eye size in tadpoles treated with gRNA sets and controls (*n* = 3 per group). (**F**) The qPCR validation of pax6 expression in the dCas9-KM-injected embryos with single gRNAs from set2 (*n* = 5 per group). All data are present as mean ± SEM. *p < 0.05, **p < 0.01, ***p < 0.001 versus gNC group. Ns, no significant differences versus gNC group

To further determine the ability of dCas9-KM system to trigger endogenous mRNA knockdown in *X. tropicalis*, we chose paired box 6 (pax6) as the second endogenous target due to its disruption affect eye development and result in a broad range of phenotypes including microphthalmia 28. We designed and generated 8 gRNAs targeting different sequences in promoter and 5’ UTR fragments of pax6 locus, and pooled them into three sets (Fig. 5A). Our data showed that co-injection of dCas9-KM mRNA with Set1 and Set2, rather than Set3, of pax6 gRNAs significantly suppressed the mRNA expression levels of pax6 at 48 hpi (Fig. 5B). Abnormal tadpoles with smaller eyes (asterisks in magnified panels) were detected in the gRNA Sets-injected groups compared with control group (Fig. 5C). The percentage of abnormal tadpoles with smaller eyes was quantified in each group and showed that injection of gRNA Sets results in about 30%~50% of tadpoles to have smaller eyes (Fig. 5D). To further support this idea, we further quantified the size of eyes in each group and found that injection of gRNA Set1 and Set2 targeting pax6 led to significant decreases in the size of eyes compared with gNC groups (Fig. 5E). To further determine which gRNA in Set2 possesses the capacity to inhibit pax6 expression, individual gRNA from Set2 was injected into embryos together with dCas9-KM. The qPCR assay revealed that pax6 mRNA expression was significantly suppressed by gP3 instead of gP5 and gP26 when compared with control group (Fig. 5F).

It has been demonstrated that the loss-of-function of T-box transcription factor T (*tbxt*) leads to compromise of notochord formation as well as a lack of posterior structures (no-tail) 29, 30. To test whether dCas9-KM system could regulate *tbxt* mRNA expression, we designed 7 gRNAs targeting *tbxt* locus and pooled them into 3 sets (Fig. S11A). The qPCR assay showed that three sets of *tbxt* gRNAs significantly suppressed the mRNA expression of *tbxt*. Among these three sets, Set3 possessed the highest efficacy to inhibit *tbxt* expression (Fig. S11B). These results imply that the development of tadpole tails might be influenced by dCas9-KM system. As expected, no-tail phenotypes were observed in three groups co-injected with *tbxt* gRNAs and dCas9-KM mRNA compared with control groups (Fig. S11C). Subsequently, we checked the potential effects of dCas9-KM system on the expression of endogenous *myh6* gene in *X. tropicalis*. Four gRNAs targeting *myh6* locus (Fig. S12A) were firstly pooled and co-injected into embryos with dCas9-KM mRNA. The qPCR assay showed that gRNA pool significantly suppressed *myh6* expression at 48 hpi (Fig. S12B). Individual gRNA injection together with dCas9-KM mRNA further revealed that gM2 and gM3 significantly reduced *myh6* expression despite gM1 and gM4 had no effects (Fig. S12C).

### Dose dependent effects of dCas9-KM system in embryos of *X. tropicalis*

**Fig. 6:**
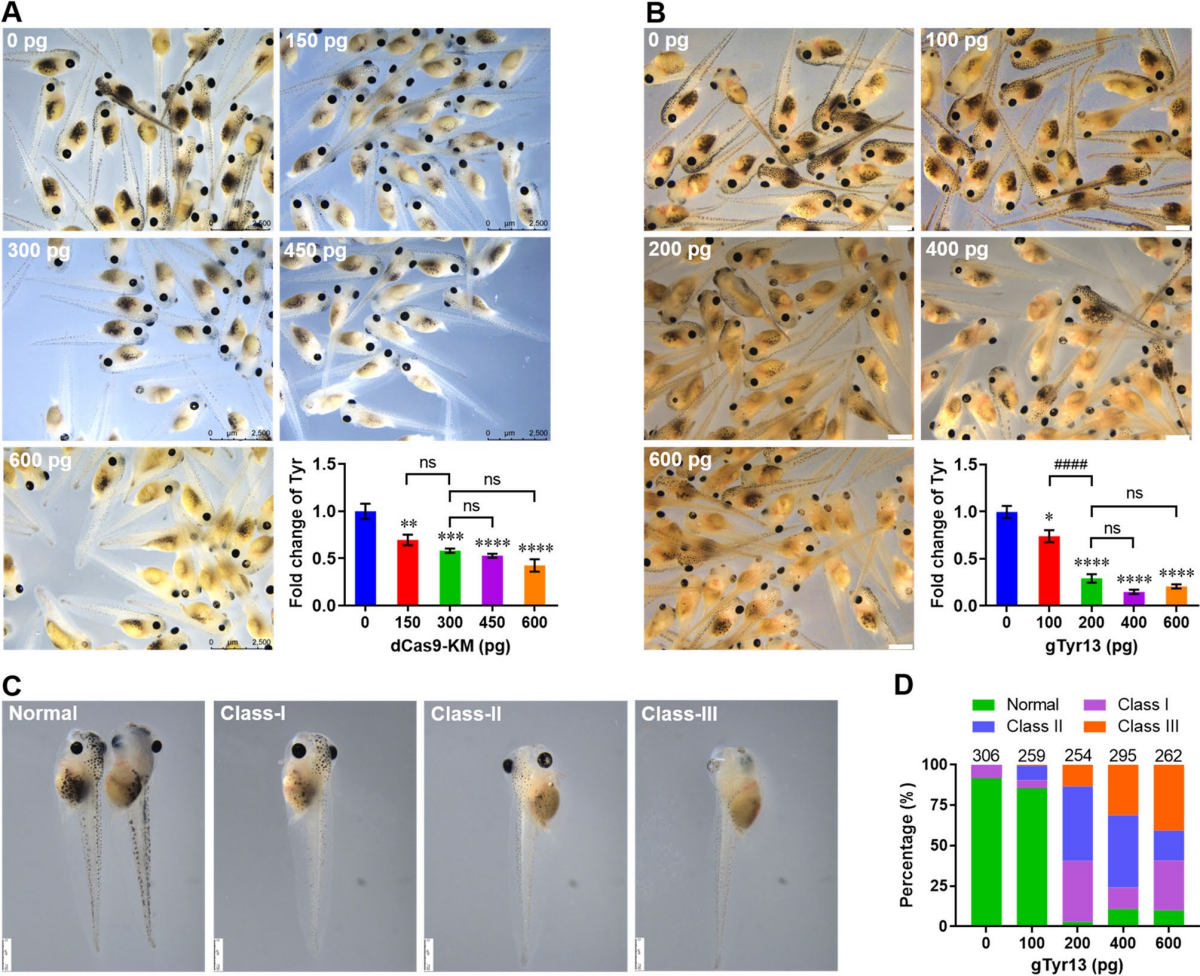
Dose effect of dCas9-KM system on the efficiency of Tyr knockdown in *X. tropicalis embryos.* (**A**) Representative images (left and upper panels) and qPCR validation (right and lower panel) of tyrosinase expression in embryos co-injected with gTry13 and dCas9-KM mRNAs at 48 hpi. Fertilized eggs were co-injected with gTry13 (200 pg/embryo) and dCas9-KM mRNAs with different concentrations from 0 to 600 pg/embryo. Data are presented as mean ± SEM (*n* = 5 per group). **p < 0.01, ***p < 0.001, ****p < 0.0001 versus control group (one-way ANOVA test). Ns, no significant differences. Scale bar = 2500 μm. (**B**) Representative images (left and upper panels) and qPCR validation (right and lower panel) of tyrosinase expression in embryos co-injected with gTry13 and dCas9-KM mRNAs at 48 hpi. Fertilized eggs were co-injected with dCas9-KM mRNAs (300 pg/embryo) and gTry13 with different concentrations from 0 to 600 pg/embryo. Data are presented as mean ± SEM (*n* = 5 per group). *p < 0.05, ****p < 0.0001 versus control group (one-way ANOVA test). ^####^*p* < 0.0001 (one-way ANOVA test). Ns, no significant differences. Scale bar = 1000 μm. (**C** and **D**) Representative images (**C**) and quantification (**D**) of embryos with different phenotypes were counted and compared with the total developed ones 48 hpi. Scale bar = 750 μm

To assess the dose dependent effects of dCas9-KM system in embryos of *X. tropicalis*, dCas9-KM mRNA and gRNA dose effects were respectively evaluated using the selected gTyr13 with highest efficacy (Fig. 4 and Fig. S9). Firstly, each embryo was co-injected with gTyr13 (200 pg) and dCas9-KM mRNA with different doses from 0 to 600 pg, followed by evaluation of *tyr* expression at 48 hpi. Representative images and qPCR assay showed that dCas9-KM mRNA could significantly suppressed the expression of *tyr* even at low dose of 150 pg compared with control. The dCas9-KM mRNA at 300 pg slightly increased the ability to reduce *tyr* expression. However, more higher doses of dCas9-KM mRNA (450 and 600 pg) did not induce significant differences in *tyr* expression compared with 300 pg (Fig. 6A). These data indicate that 300 pg dCas9-KM mRNA is enough to trigger specific endogenous mRNA in *X. tropicalis*. Therefore, different dose of gTyr13 (0-600 pg/embryo) were then co-injected into embryo with 300 pg dCas9-KM mRNA to evaluate the dose effect of gRNA. We found that 100 pg gTyr13 could moderately suppressed *tyr* expression. However, 200 pg gTyr13 greatly increased the efficacy of dCas9-KM system to inhibit *tyr* expression compared with 100 pg. Further increases in gTyr13 dose (400 and 600 pg/embryo) did not significantly induce suppression of *tyr* expression compared with 200 pg (Fig. 6B). However, quantification of albinism phenotypes revealed that high doses of gTyr13 (400 and 600 pg/embryo) resulted in a high increase in the percentage of severe albinism phenotypes (Class III) compared with middle dose of gTyr13 at 200 pg/embryo (Fig. 6C and D). Moreover, severe albinism tadpoles with complete ablation of pigmentation in dual eyes and body were observed in 600 pg instead of 400 pg gTyr13 group (Fig. S13).

### Effects of regulators on dCas9-KM system

The coat protein of the MS2 phage (MCP) binds as a dimer to a small RNA stem-loop (MS2-aptamer) structure with high affinity. This interaction has been used in the regulation of gene expression. It has been demonstrated that MS2-aptamer modified gRNA (MS2-gRNA) combined with MCP-VP64 fusion can enhance the basal dCas9-VP64 activity to promote transcription 31. In addition, the MS2-MCP platform has been used in dCas9 system for live cell imaging by generating chimaeric transcripts of MS2-gRNA, which when co-expressed with dCas9 can recruit fluorescently tagged MCP 32. These previous reports indicated that MS2-gRNA can recruit dCas9- and MCP-tagged regulators to specific genomic sites. To determine whether MS2-MCP platform can increase the activity of dCas9-KM system, dCas9 and MCP were fused with transcription repressor KRAB (dCas9-KRAB, dCas9-K) and MeCP2 (MCP-MeCP2), respectively. The MS2-gTyr13 or MS2-gNC were co-injected into *X. tropicalis* fertilized eggs at one-cell stage with dCas9-K and MCP-MeCP mRNAs (Fig. S14A). Our data showed that MS2-gTyr13 significantly reduced *tyr* mRNA expression by about 50% at 48 hpi compared with MS2-gNC (Fig. S14B). Albinism phenotype quantification showed that about 50% tadpoles with pigmentation ablation in MS2-gTry13-injected group compared with MS2-gNC (Fig. S14C and D).

**Fig. 7:**
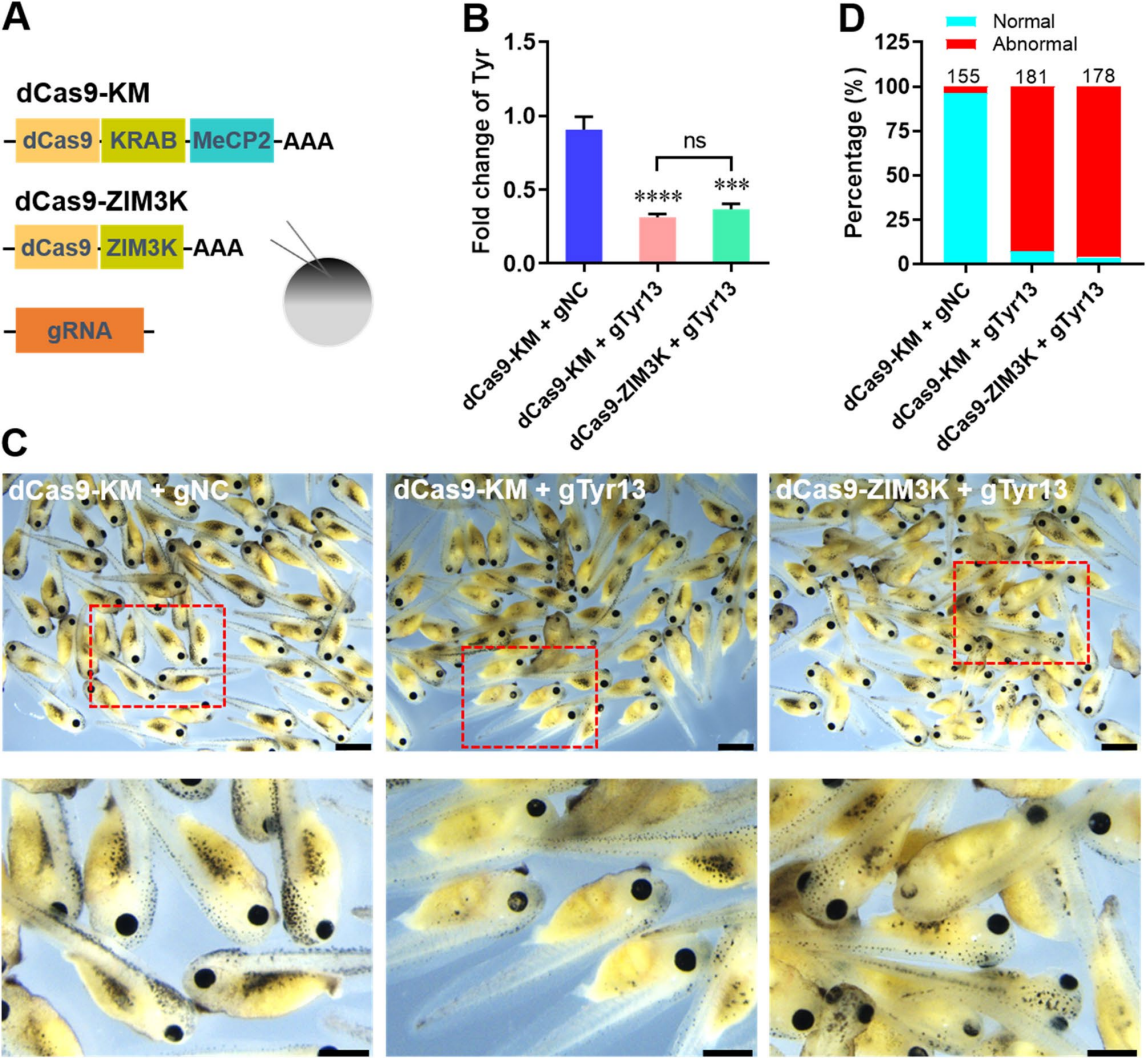
Effects of ZIM3-KRAB domain on CRISPRi-induced tyr knockdown in embryos of *X. tropicalis*. (**A**) Schematic illustration of the experimental setup used to analyze the capacity of KRAB domain from Zim3 gene (ZIM3K) to target exogenous reporter gene in *X. tropicalis* embryos. The gTry13 were injected into the fertilized eggs together with dCas9-KM or dCas9-ZIM3K mRNAs, followed by embryo evaluation at 48 hpi as follows. (**B**) The qPCR validation of Tyr expression in the injected embryos. Data are presented as mean ± SEM (*n* = 5 per group). ****p < 0.0001 versus control group (one-way ANOVA test). Ns, no significant differences. (**C**) Representative images of tyrosinase expression in embryos co-injected with gTry13 and dCas9-mediated repressors at 48 hpi. The top panel shows low magnification (Scale bar = 1 mm), and the lower panel shows high magnification (Scale bar = 500 μm). (**D**) Embryos with different phenotypes were counted and compared with the total developed ones after injection. Total embryos evaluated for each group (*n*) is shown above each column

CRISPRi system based on dCas9 fused to KRAB transcriptional repression domain (dCas9-KRAB, dCas9-K) has been widely adopted as a genetic engineering tool 21, 24, 33. The KRAB domain from KOX1 is the most wildly used repressor in CRISPRi system due to it was the first functionally characterized KRAB 22. Subsequently, the methyl-CpG binding protein 2 (MeCP2) was found to enhance the repressive activity of dCas9-K system in mammalian cells by fusing to KARB (dCas9-KRAB-MeCP2, dCas9-KM) 27. Recently, it has been demonstrated that a new KRAB domain from ZIM3 protein (ZIM3-KRAB, ZIM3K) alone was a stronger repressor than the bipartite KRAB-MeCP2 in human cells 34. To determine the effects of ZIM3K domain on traditional dCas9-KM system in *X. tropicalis*, the ZIM3K was fused to dCas9 (dCas9-ZIM3K), which was compared with dCas9-KM system. The gTyr13 was co-injected into fertilized eggs at one-cell stage with dCas9-KM and dCas9-ZIM3K mRNAs, respectively (Fig. 7A). The qPCR assay showed that dCas9-KM and dCas9-ZIM3K mRNA injection together with gTyr13 greatly suppressed the mRNA expression of *tyr* at 48 hpi when compared with dCas9-KM/gNC group (Fig. 7B). Moreover, there was no significant differences in *tyr* expression levels between dCas9-KM and dCas9-ZIM3K group, indicating that dCas9-ZIM3K possesses the comparable repressive activity with dCas9-KM system in *X. tropicalis* (Fig. 7B). As expected, tadpole images showed that great ablation of pigmentation was detected in dCas9-KM and dCas9-ZIM3K groups (Fig. 7C). In agreement with these results, quantification of phenotype revealed that both dCas9-KM and dCas9-ZIM3K systems resulted in more than 90% tadpoles with abnormal pigmentation compared with control group (Fig. 7D).

## Discussion

The *X. tropicalis* is an ideal and important vertebrate animal model for genetics, developmental and regenerative biology, due to its diploid genetic background and short generation time. The gene disruption and modification in DNA levels has been well established using CRISPR-Cas9 system in *X. tropicalis*^3^–^5^. However, this strategy needs a multi-generation genotype screening to establish a permanent genetic strain for exploring specific gene function in *X. tropicalis*. In the present study, we systematically examined the potential of CRISPR-Cas13 and CRISPRi systems to target mRNA transcripts in *X. tropicalis*. We showed that CRISPRi rather than CRISPR-Cas13 is a robust and efficient method to suppress mRNA expression in *X. tropicalis* embryos, and proposed that CRISPRi composed of dCas9-KM or dCas9-ZIM3K is a suitable knockdown platform for silencing gene expression *X. tropicalis* embryos.

RNA knockdown is a promising strategy that allows for temporary and non-heritable silencing of target genes. RNA-targeting CRISPR-Cas13 systems have been employed for a variety of applications including RNA knockdown 35. It has been reported that Cas13 systems have high RNA knockdown capacity in many organisms including plants, zebrafish, and mammalian cells 12–17. Previous studies have compared the RNA targeting ability of Cas13a, Cas13b, and Cas13d to each other and to RNAi targeting the same transcript target sequence. These three Cas13 proteins have been reported to be superior at specifically targeting RNA compared to RNAi 12, 14, 18. Using luciferase reporter assay and position-matched gRNAs, it has been demonstrated that PspCas13b possesses greatly increased knockdown potential relative to LwaCas13a in mammalian cells 12. However, RfxCas13d can mediate significantly greater transcript knockdown than both LwaCas13a and PspCas13b in mammalian cells using position-matched gRNAs 14. We thus evaluated the RNA targeting capacities of PspCas13b and five Cas13d variants (RfxCas13d, AdmCas13d, EsCas13d, P1E0Cas13d, RffCas13d) in *X. tropicalis* embryos. Our data showed that PspCas13b failed to reduce reporter gene expression in *X. tropicalis* (Fig. 1E-G). Moreover, no significantly decreases in reporter gene expression were detected in *X. tropicalis* embryos injected with Cas13d variants including RfxCas13d (Fig. 1H-L and Fig. S2). In addition, we did not observe a significant interference of endogenous tyrosinase gene in *X. tropicalis* embryos for PspCas13b and RfxCas13d system (Fig. 2). These findings suggest that Cas13 systems might be not suitable for RNA knockdown in the embryos of *X. tropicalis*, even they can mediate high capacity for RNA knockdown in other organisms. These different results may be induced by an unknown species-specific mechanism in *Xenopus* compared with other organisms. This conjecture can be partially supported by the fact that RNAi is not work in *Xenopus* due to the limiting Ago2 protein levels, although it can robustly and efficiently target RNA knockdown in other species 6.

CRISPRi is another powerful platform to inhibit transcription of target genes through dCas9 and gRNA complex, which is reported in early 2013 for the first time 21. It was much more valuable when the transcription inhibitor was fused with dCas9. Previous reports showed that the fusion of dCas9 with the KRAB transcriptional repression domain from KOX1 protein (dCas9-KRAB) significantly enhances the capacity of CRISPRi system based on dCas9 alone in human cells 23–25. However, the dCas9-KRAB system suffers from inefficient knockdown and poor performance when compared with Cas9 nuclease-based methods 36. More recently, it was reported that the fusion of TRD domain of MeCP2 with dCas9-KRAB repressor (dCas9-KRAB-MeCP2, dCas9-KM) can greatly increase the capacity of CRISPRi to target gene expression in human cells 27. Indeed, the improved RNA knockdown capacity of dCas9-KM system were further confirmed in human induced pluripotent stem cells 37, primary rat neurons 38, hamster ovary cells 39, and chicken DF-1 cells 40. The CRISPRi based on dCas9-KM fusion protein provided an efficient and specific genome targeting platform that could knock down RNA transcription without changing the target DNA sequence. Given that Cas9-mediated genome editing has been well and efficiently employed in *Xenopus*^3^–^5^, we speculate that dCas9-KM system might be another potential approach for targeting RNA knockdown in *X. tropicalis*. As expected, our data showed that exogeneous and endogenous transcripts can be efficiently targeted by CRISPRi based on dCas9-KM system, resulting in a significant and great decrease in transcript levels (Figs. 3, 4 and 5). Through comparing studies, we found that dCas9-KM system possesses most high capacity to suppress target gene expression in *X. tropicalis* embryos, followed by dCas9-K and dCas9 alone (Fig. S8). Although we did not define the precise effective duration of CRISPRi in *X. tropicalis* embryos, our data reveal that CRISPRi-induced knockdown of endogenous target gene can maintain 4 ~ 5 days, as demonstrated by the fact that dCas9-KM-induced pigmentation loss was slightly recovered at 5 dpi, followed by pigmentation restoration at 9 dpi. More recently, a new KRAB domain from ZIM3 protein (ZIM3-KRAB, ZIM3K) was identified as an exceptionally potent repressor. Moreover, the ZIM3K domain alone was a stronger repressor in human cells than the bipartite KRAB-MeCP2 fusion based on the KRAB domain from Kox1 gene 34. In the present study, we found that the new ZIM3K domain alone has a comparable rather than stronger capacity for targeting transcripts in *X. tropicalis* when compared with the bipartite KRAB-MeCP2 repressor based on the KRAB domain from Kox1 gene (Fig. 7).

Taken together, our findings propose that CRISPRi rather than CRISPR-Cas13 system is an efficient and direct approach for specific gene function study during embryogenesis of *X. tropicalis* by targeting transcripts. Moreover, the CRISPRi systems based on dCas9-ZIM3K and dCas9-KM possess comparable knockdown capacities in *X. tropicalis*, which are suitable platforms to efficiently suppress specific gene expression in *X. tropicalis*. It will further extend the application of *X. tropicalis* animal model for specific gene function exploring during organ development and regeneration.

## Methods

### Plasmids

The human codon-optimized PspCas13b (pC0046, #103862) and RfxCas13d (pXR001, #109049) expressing plasmids as well as their guide RNA (gRNA) expression plasmids (pC0043, #103854 for PspCas13b; pXR003, #109053 for RfxCas13d) were purchased from Addgene. The NLS-RfxCas13d-NLS fragment in pXR001 plasmid was amplified and subcloned into the multiple clone site (MCS) of pcDNA3.1 plasmid (#ZT119) (FENGHUISHENGWU, China) to construct pcDNA3.1-RfxCas13d plasmid. The pEGFP-N1 (#BR083) (FENGHUISHENGWU, China) plasmid was used as a reporter system in human cells to evaluate Cas13 capacity. The gRNAs targeting EGFP were designed according to CRISPR-Cas13 targeting data in mammalian cell culture in which there was not any significant protospacer flanking site preference (PFS) reported and gRNA activity partially correlated with target accessibility 12, 14. The gRNA targeting LacZ 12 was used as the negative control (gNC). Designed gRNAs were cloned into pC0043 and pXR003 plasmids, respectively.

The pmCherry-N1 plasmid (#BR049) (FENGHUISHENGWU, China) was used as a reporter system in *X. tropicalis* embryos to evaluate CRISPRi capacity. Moreover, a mCherry-bGH-poly(A)-EF1α fragment was synthesized by Generay Biotech (Shanghai, China) and subcloned into the MCS of pEGFP-N1 plasmid to construct pCMV-mCherry-EF1α-EGFP plasmid, which can be used a dual-reporter system in *X. tropicalis* embryos for CRISPRi evaluation.

### Cell culture and transfection

HEK293T cells were cultured in DMEM medium (Gibco, USA) containing 10% fetal bovine serum (FBS) (Gibco, USA) and 0.5% streptomycin/penicillin solution (10,000 U/ml, Invitrogen), at 37 °C in a 5% CO_2_ incubator. For transient transfection experiment, cells were seeded in 24-well plate at a density of 5 × 10^4^ cells/well. After overnight incubation, cells were transfected by PspCas13 system (pC0046 and pC0043) or RfxCas13d system (pcDNA3.1-RfxCas13d and pXR003) together with pEGFP-N1 plasmid, using the LipoFiterTM Liposomal Transfection Reagent (Hanbio Biotechnology, Shanghai, China). The culture media were changed at 8 h post transfection, and the transfected cells were cultured until 48 h. Fluorescence of EGFP reporter gene was detected by a fluorescent microscope (Olympus, Tokyo, Japan) at 488 nm. Quantitative expression of reporter gene was examined by flow cytometry on a CytoFLEX flow cytometer (Beckman Coulter) and data analysis was performed using CytExpert 2.0 software (Beckman Coulter).

### Production of mRNA and gRNA

The *X. tropicalis* codon-optimized PspCas13b 12 and Cas13d orthologs (RfxCas13d, AdmCas13d, EsCas13d, p1E0Cas13d, and RffCas13d) 14 were synthesized by Generay Biotech (Shanghai, China) and cloned into the pCS2 + vector 41. These constructs were linearized and transcribed with mMessage mMachine SP6 Kit (Ambion) and purified with the RNeasy Mini Kit (Qiagen) to generate capped Cas13 mRNAs. The capped EGFP and mCherry mRNAs were used as reporter and internal control in *X. tropicalis* embryos, respectively. Cas13-related gRNAs targeting EGFP (gEGFP), tyrosinase (gTyr), and LacZ (gNC) were designed using RNAfold software (http://rna.tbi.univie.ac.at//cgi-bin/RNAWebSuite/RNAfold.cgi). For all the Cas13 gRNA transcription, oligonucleotides carrying the T7 promoter and appropriate downstream sequence were synthesized (Generay Biotech, Shanghai, China) and annealed with an antisense T7 oligo for gRNAs and PCR-amplified for target and array templates 14. The oligo anneal and PCR templates were in vitro transcribed with the Transcript Aid T7 High Yield Transcription Kit (Thermo Fisher Scientific) and purified with the miRNeasy Mini Kit (Qiagen) as previously reported 16. The gRNA, scaffold, and primer sequences are listed in Supplementary Table 1. Moreover, the codon-optimized PspCas13b and RfxCas13d fragments were also subcloned into the *Xenopus* transgenic plasmid under control of the ubiquitous CAG promoter to overexpress them in embryos 42.

For CRISPRi systems, the *X. tropicalis* codon-optimized dCas9, dCas9-KRAB (dCas9-K, the KRAB domain from Kox1 gene), dCas9-KRAB-MeCP2 (dCas9-KM) 27, MCP-MeCP2, and dCas9-ZIM3K (ZIM3K, the KRAB domain from Zim3 gene) 34 were synthesized by Generay Biotech (Shanghai, China) and cloned into the pCS2 + vector 41. These constructs were linearized and transcribed with mMessage mMachine SP6 Kit (Ambion) and purified with the RNeasy Mini Kit (Qiagen) to generate capped dCas9-related mRNAs. Capped mCherry and EGFP mRNAs were used as reporter and internal control in *X. tropicalis*, respectively. The DNA fragment of target gene, 500 bases upstream and 300 bases downstream of the TSS that conform with the nucleotide requirements for transcription, was used to search candidate gRNAs using the CRISPR/Cas9 online predictor CCTop (https://cctop.cos.uni-heidelberg.de/) as previously described 43. The gRNA targeting LacZ was used as negative control (gNC). For the dCas9 gRNA in vitro transcription, all designed gRNAs were firstly cloned into the pUC57-Simple-gRNA backbone 4. For MS2-gRNA generation, the pUC57-gRNA-MS2 plasmid containing MS2 stem loops inserted into the tetraloop and stem loop 2 of gRNA was synthesized by Generay Biotech (Shanghai, China) according to the sgRNA2.0 sequence 44. The in vitro transcription of gRNAs were generated by the Transcript Aid T7 High Yield Transcription Kit (Thermo Fisher Scientific) and purified with the miRNeasy Mini Kit (Qiagen) as previously reported 5. The gRNA and scaffold sequences are listed in Supplementary Table 2.

### Embryo manipulation, injection and image acquisition

The *X. tropicalis* frogs were purchased from Nasco (USA) and bred in an in-house facility. Embryos were obtained by in vitro fertilization as previously reported 45. The use of *X. tropicalis* was approved by the Institutional Animal Care and Use Committee of Jinan University. Cas13- or dCas9-related mRNA and their corresponding gRNA were co-injected into the animal pole of *X. tropicalis* embryos (fertilized eggs) at one-cell stage. For exogenous reporter gene assay, the reporter and internal control mRNA were simultaneously injected. Each embryo was injected with 2 nl solution containing Cas13- or dCas9-related mRNA (300 pg) and gRNA (100 pg) with or without reporter and internal control (each 100 pg). The injected embryos were raised for further analysis in indicated time points. Phenotypes and samples were collected, analyzed and quantified between 15 h and 9 days post injection depending on different experiments. The *X. tropicalis* embryo phenotypes and fluorescent images were capture by fluorescence stereomicroscope (Leica M205FA). EGFP and mCherry fluorescence were quantified using Image-Pro Plus version 6.0 software (Media Cybernetics, Bethesda, MD). EGFP and mCherry fluorescence was subtracted to un-injected embryos and then final values were calculated.

### Quantitative real-time PCR (qPCR)

Total RNA was isolated from *X. tropicalis* embryos at indicated developmental time points using TRI Reagent (Molecular Research Center Inc., USA). cDNA was synthesized from total RNA (2 µg) and oligo (dT)18 primers (0.5 µg) using the ReverTra Ace^®^ qPCR RT Kit (Toyobo, Japan). The qPCR analysis was performed using QuantiTect SYBR green PCR Master Mix (Qiagen GmbH, Hilden, Germany) and the MiniOpticon Real-Time PCR System (Bio-Rad, CA, USA). After denaturation for 10 min at 95 °C, the reactions were subjected to 45 cycles of 95 °C for 30 s, 60 °C for 30 s, and 72 °C for 30 s. *Odc* was used as the internal standard control to normalize the expression of endogenous target genes using the △△Ct method. The qPCR primer sequences are listed in Supplementary Table 3. In the present study, the stabilization of internal standard *odc* was proved by its stable Ct values in *X. tropicalis* embryos treated with dCas9-KM system and different concentrations of gTyr13 (Supplementary Table 4).

### Statistical analysis

All statistics were calculated using GraphPad Prism 8 Software. All data are presented as the mean ± SEM. Unless otherwise specified, the number (*n*) used for statistical analysis denotes the independent experiments. Among three or more groups, statistical analysis was performed using one-way ANOVA followed by Dunnett’s multiple comparison tests. Comparisons between two groups were analyzed using unpaired Student’s *t*-test. A *p* value of less than 0.05 was considered statistically significant.

## Electronic supplementary material

Below is the link to the electronic supplementary material.

Supplementary Material 1: Supplementary figures 1-14

Supplementary Material 2: Supplementary tables 1-4

## Data Availability

The data that support the findings of this study are available from the corresponding author upon reasonable request.
