# Supplementary material for: CRISPR/Cas-mediated mRNA knockdown in the embryos of Xenopus tropicalis
Source: Cell Biosci. 2025 Apr 23;15:52. doi: 10.1186/s13578-025-01397-8 (PMC12020200; doi:10.1186/s13578-025-01397-8)
Supplement: Supplementary file 1 — Supplementary Material 1: Supplementary figures 1-14 [file 13578_2025_1397_MOESM1_ESM.docx]

**Supplementary materials**

**CRISPR/Cas-mediated mRNA knockdown in the embryos of *Xenopus tropicalis***

Supplementary figures:1-14

**Supplementary figures and figure legends**

**
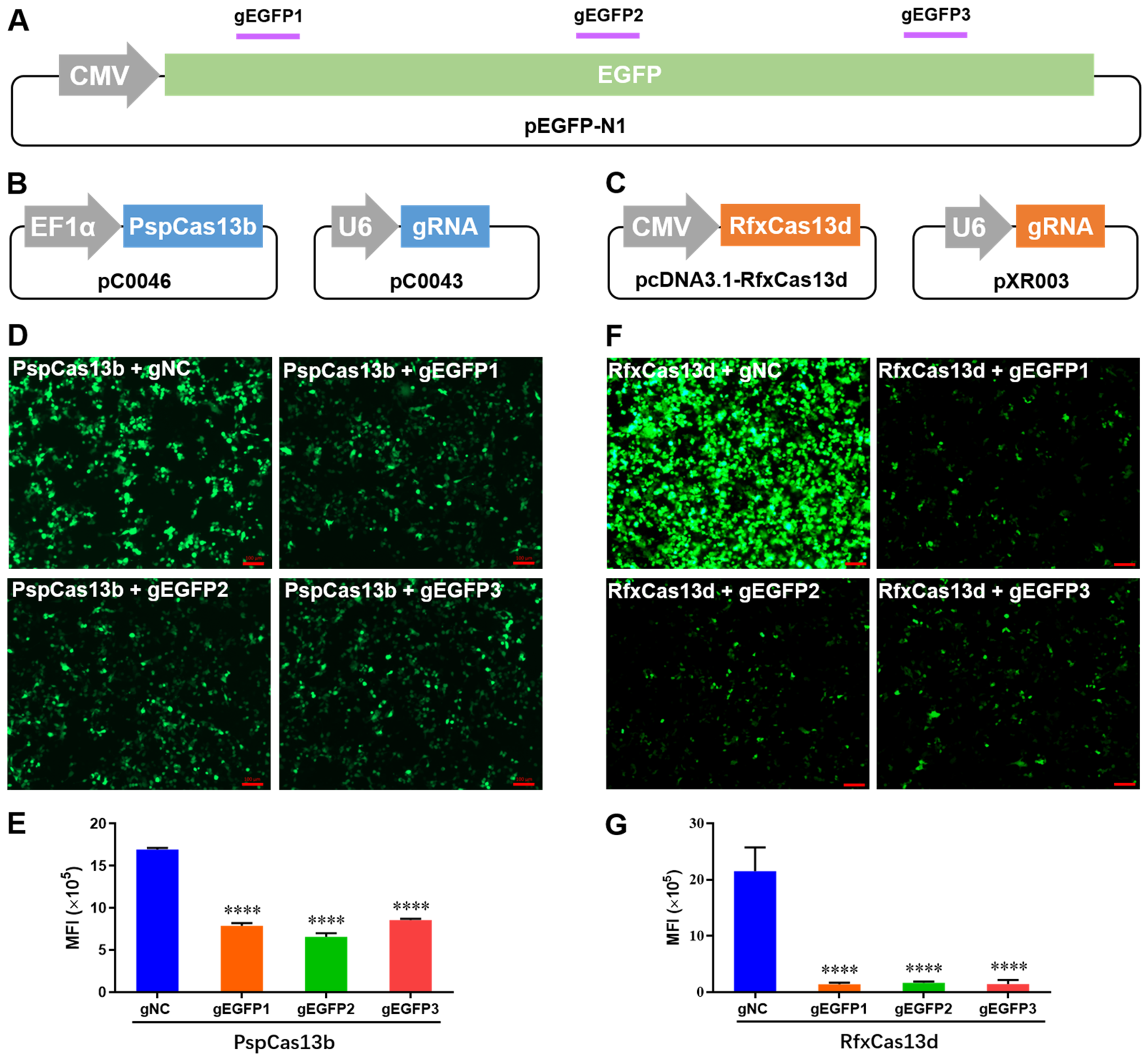
**

**Figure S1. Effects of PspCas13b and RfxCas13d systems on reporter gene expression in mammalian cells.** (A) Schematic of Cas13-related gRNAs targeting the mRNA of EGFP reporter gene in pEGFP-N1 plasmid. (B and C) Schematic plasmid maps of PspCas13b (B) and RfxCas13d (C) system used in human cells. (D and E) Representative images (D) and quantification (E) of EGFP fluorescence in 293T cells transfected with PspCas13b system. Data are presented as mean ± SEM (*n*=5), *****p*<0.0001 (one-way ANOVA test). Scale bar=100 µm. (F and G) Representative images (F) and quantification (G) of EGFP fluorescence in 293T cells transfected with RfxCas13d system. Data are presented as mean ± SEM (*n*=5), *****p*<0.0001 (one-way ANOVA test). Scale bar=100 µm.


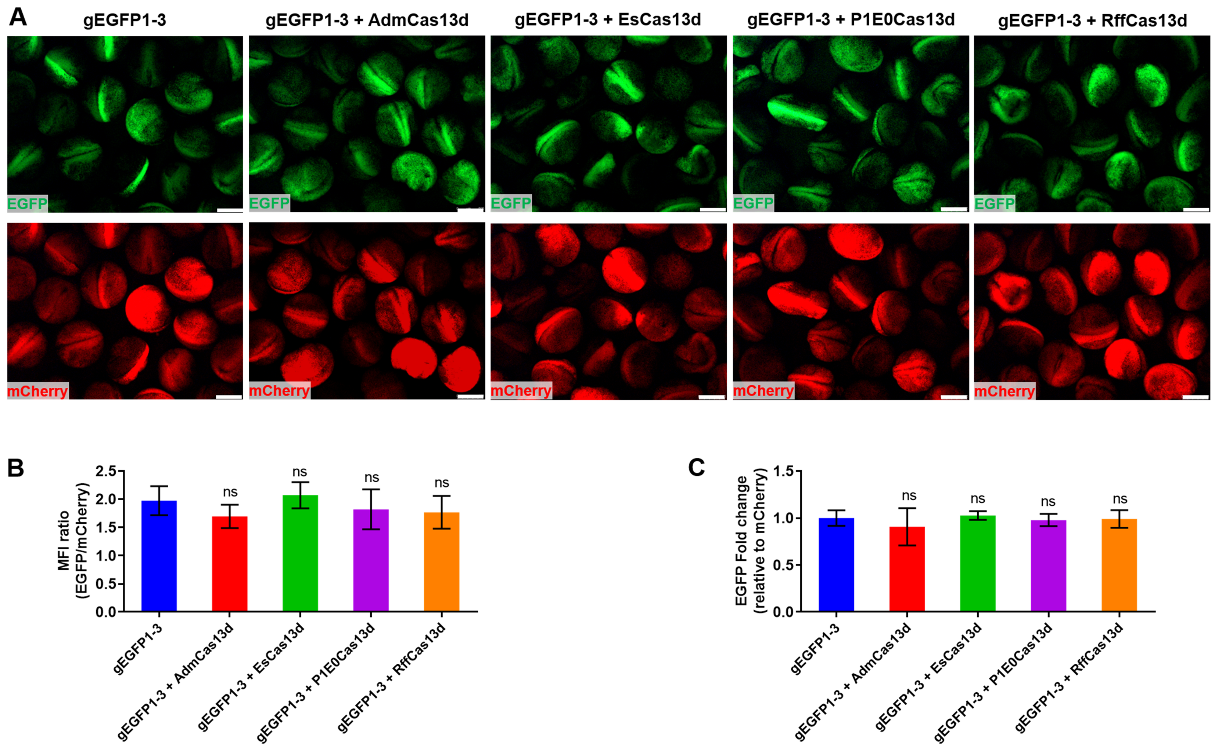


**Figure S2. Effects of other Cas13d variants on reporter gene expression in embryos of *X. tropicalis*.** (A) Representative images of EGFP fluorescence in control and experimental embryos injected with other Cas13d variants at 15 hpi. The mCherry was used as the internal control. Scale bar=500 µm. (B) Quantification of the mean fluorescent intensity (MFI) ratio (EGFP/mCherry) in control and experimental embryos injected with other Cas13d variants at 15 hpi. (C) The qPCR validation of EGFP expression in control and experimental embryos injected with other Cas13d variants at 15 hpi. The mCherry was used the internal control. Data are presented as mean ± SEM (*n* =~30 embryos from 3 independent experiments), one-way ANOVA test. Ns, no significant differences versus control.


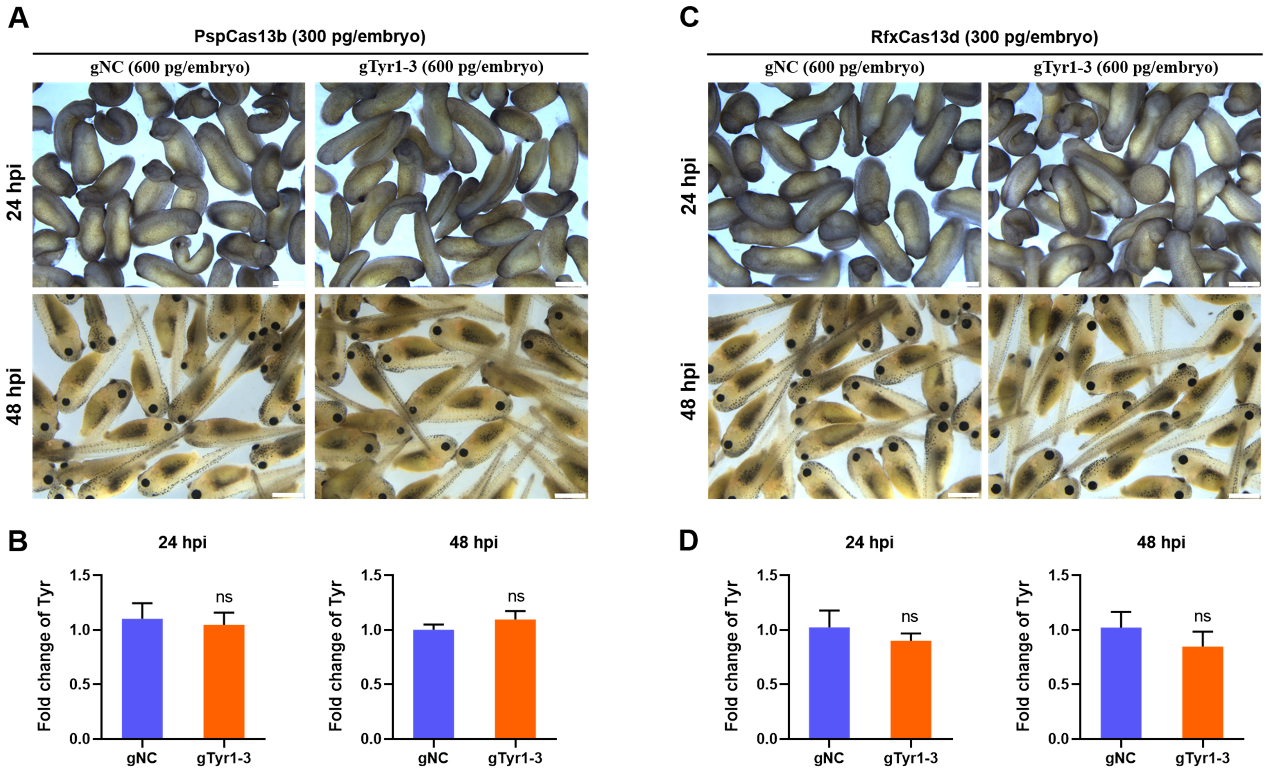


**Figure S3. Effects of Cas13 system combined with high dose of gRNAs on *tyr* expression in embryos of *X. tropicalis*.** Embryos were co-injected with high dose of gTyr1-3 (600 pg/embryo) and Cas13 mRNAs (300 pg/embryo) for 24 and 48 hours, respectively. (A and B) Representative images (A) and quantification (B) of *tyr* expression in control and experimental embryos injected with PspCas13b system at 24 and 48 hpi (*n*=3). (C and D) Representative images (C) and quantification (D) of *tyr* expression in control and experimental embryos injected with RfxCas13d system at 24 and 48 hpi (*n*=3). Data are presented as mean ± SEM. Scale bar=750 µm. Ns, no significant differences versus control.


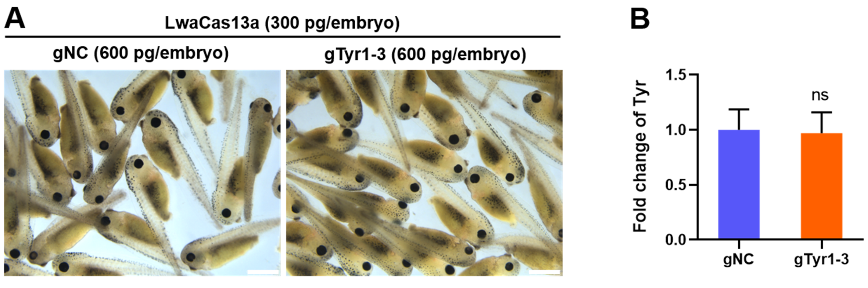


**Figure S4. Effects of gTyr1-3 set combined with LwaCas13a on *tyr* expression in embryos of *X. tropicalis*.** Embryos were co-injected with high dose of gTyr1-3 (600 pg/embryo) and LwaCas13a mRNAs (300 pg/embryo) for 48 hours. (A and B) Representative images (A) and quantification (B) of *tyr* expression in control and experimental embryos injected with LwaCas13a system at 48 hpi (*n*=3). Data are presented as mean ± SEM. Scale bar=750 µm. Ns, no significant differences versus control.


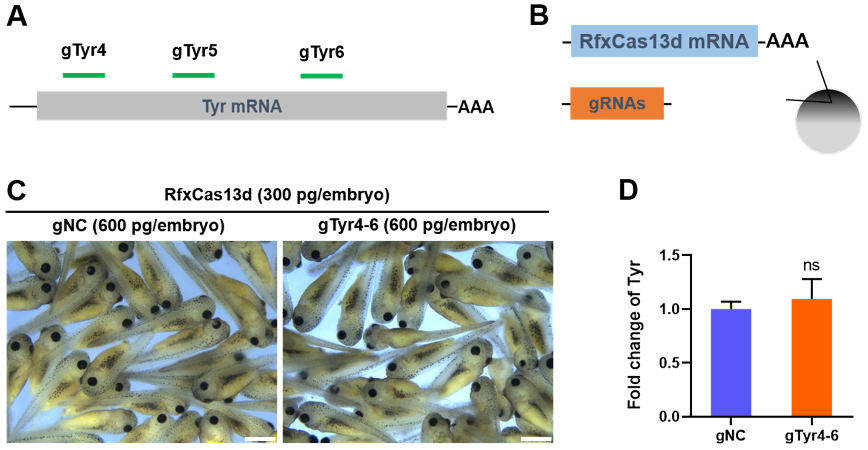


**Figure S5. Effects of gTyr4-6 set combined with RfxCas13d on *tyr* expression in embryos of *X. tropicalis*.** (A) Schematic of RfxCas13d-related gRNA4-6 targeting the mRNA of *tyr* gene. (B) Schematic illustration of the experimental setup used to analyze the capacities of RfxCas13d together with gTyr4-6. (C and D) Representative images (C) and quantification (D) of *tyr* expression in control and experimental embryos injected with RfxCas13d system at 48 hpi (*n*=3). Data are presented as mean ± SEM. Scale bar=750 µm. Ns, no significant differences versus control.


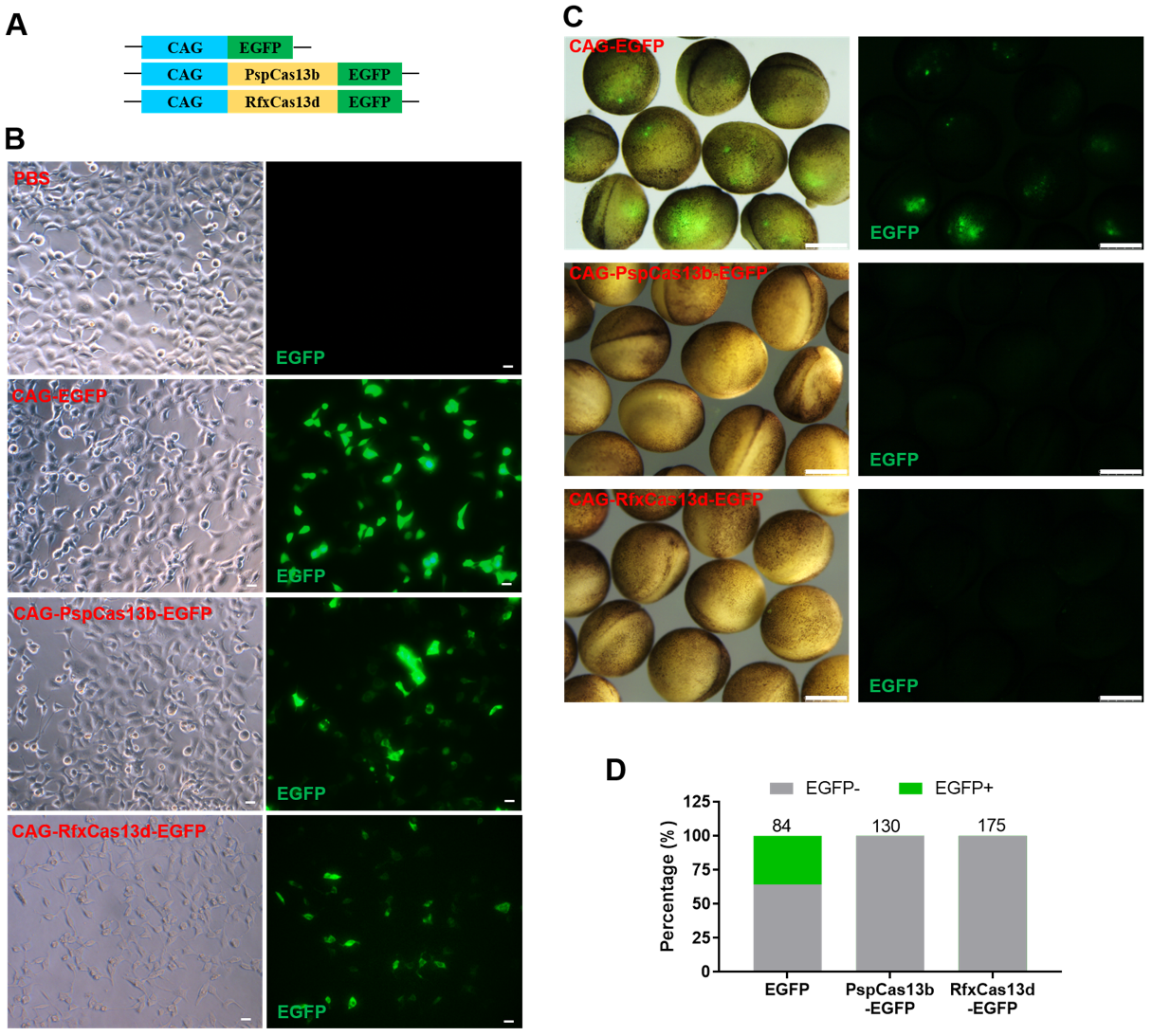


**Figure S6. Expression of Cas13 proteins in mammalian cells and *X. tropicalis* embryos.** (A) Schematic of plasmids harboring the expression cassette of Cas13-EGFP fusion protein. The pCAG-EGFP plasmid is used as a positive control. (B) Representative images of EGFP expression in 293T cells transfected with pCAG-EGFP, pCAG-PspCas13b-EGFP, and pCAG-RfxCas13d-EGFP plasmids for 48 hours. PBS was used as a negative control. Scale bar=50 µm. (C and D) Representative images (C) and quantification of embryos with EGFP expression were counted and compared with the total developed ones 15 hours post injection of indicated plasmids. Scale bar=500 µm. Total embryos evaluated for each group (n) is shown above each column.


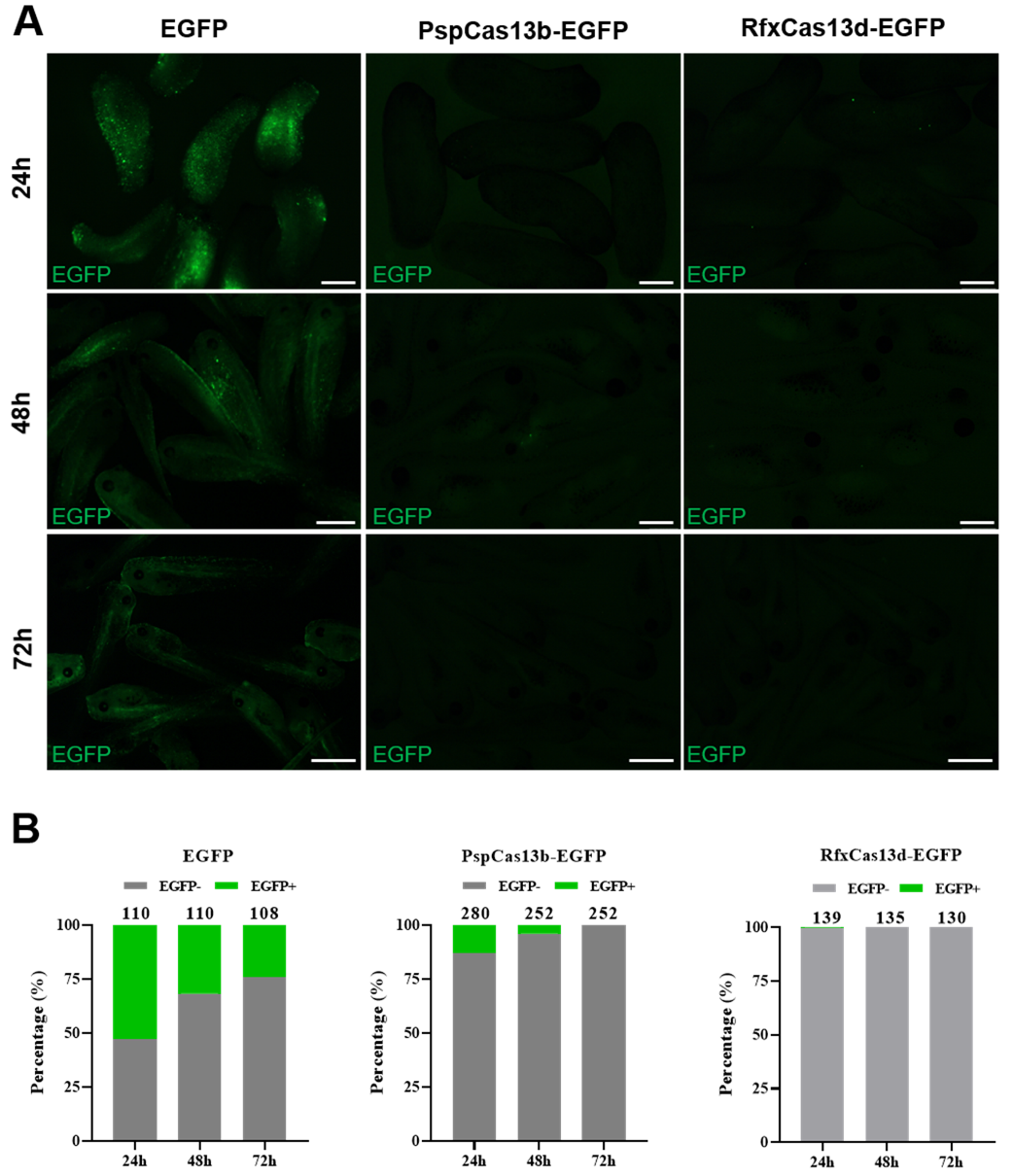


**Figure S7. Expression of Cas13 proteins in *X. tropicalis* embryos injected with mRNAs.** *X. tropicalis* embryos were injected with mRNAs of EGFP, PspCas13b-EGFP, and RfxCas13d-EGFP, followed by determination of EGFP signals at 24~72 h post-injection. (A) Representative images of EGFP expression in embryos injected with EGFP, PspCas13b-EGFP, and RfxCas13d-EGFP mRNAs at indicated time points. Scale bar=500 µm. (B) Quantification of embryos with EGFP expression were counted and compared with the total developed ones at indicate time points. Total embryos evaluated for each group (n) is shown above each column.

**
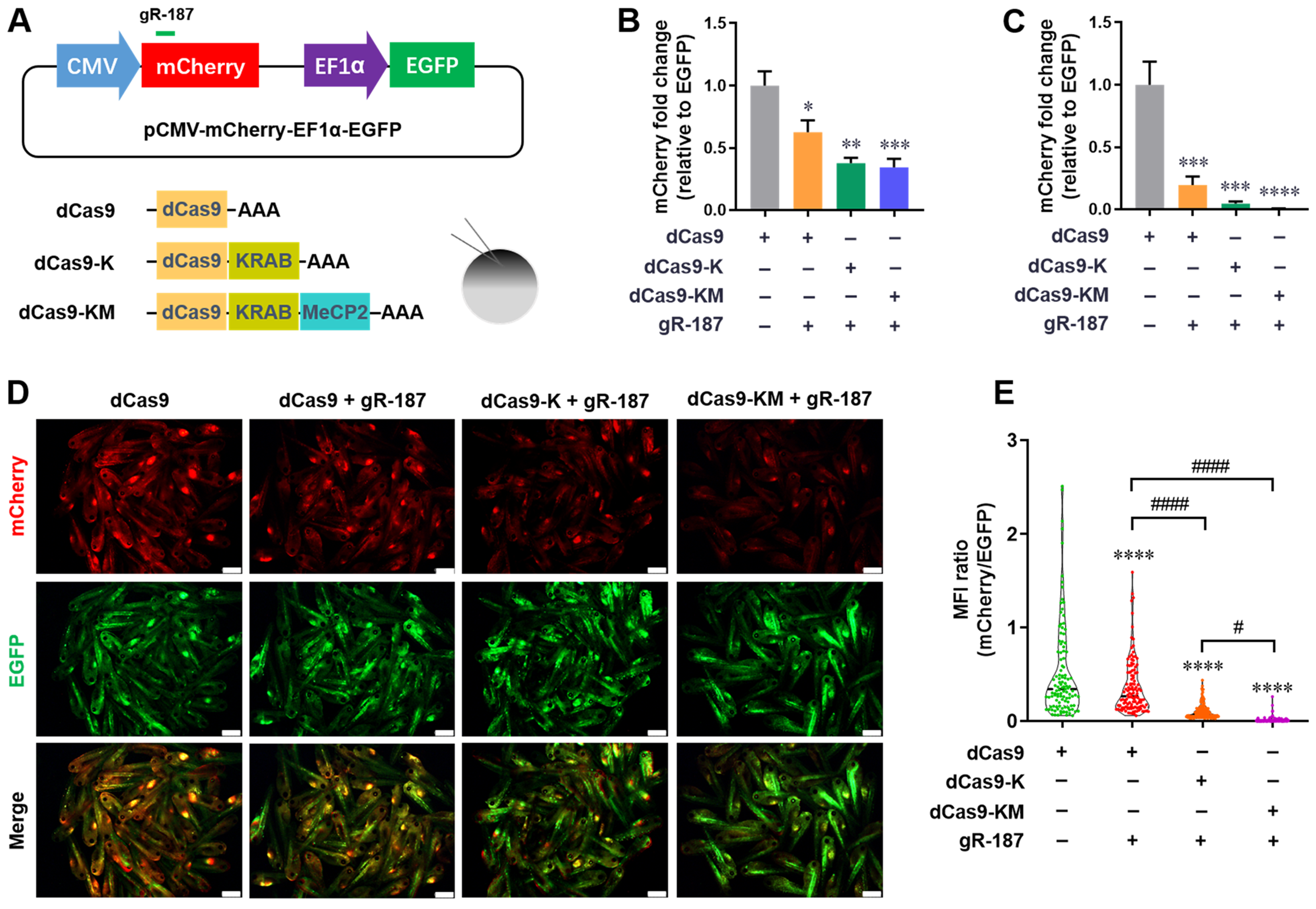
**

**Figure S8. Effects of different CRISPRi effectors on reporter gene expression in embryos of *X. tropicalis*.** (A) Schematic illustration of dual-reporter plasmid (upper panel) and the experimental setup used to analyze different CRISPRi effectors in *X. tropicalis* embryos (lower panel). (B and C) The qPCR validation of mCherry expression in embryos co-injected with different CRISPRi effector mRNAs and indicated gRNA at 24 (B) and 48 (C) dpi. EGFP was used as the internal control. Data are presented as mean ± SEM (*n*=5 per group), **p*<0.05, ***p*<0.01, ****p*<0.001, *****p*<0.0001 versus dCas9 alone. one-way ANOVA test. (D and E) Representative images (D) and quantification (E) of mCherry expression in embryos co-injected with different CRISPRi effector mRNAs and indicated gRNA at 48 hpi. Data are presented as mean ± SEM (*n*=~120 embryos from 3 independent experiments), *****p*<0.0001 versus dCas9 alone, ^#^*p*<0.05, ^####^*p*<0.0001 (one-way ANOVA test). Scale bar=1 mm.


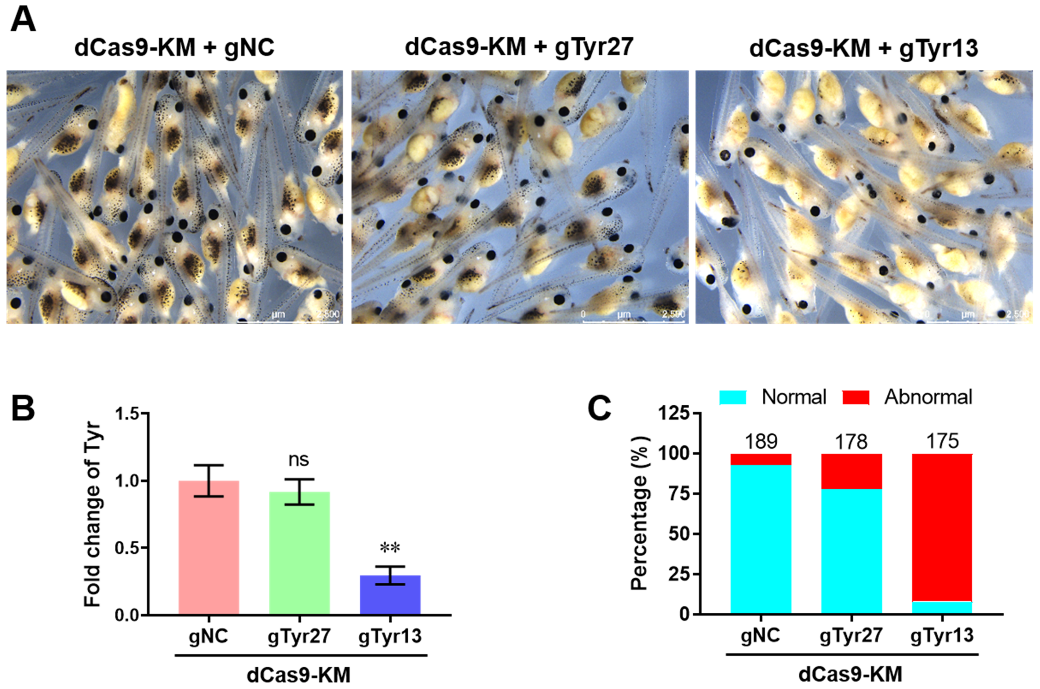


**Figure S9. Comparation of Tyr knockdown efficiency between gTyr27- and gTyr13-mediated CRISPRi.** The dCas9-KM mRNA was co-injected into the animal pole of *X. tropicalis* fertilized eggs at one-cell stage with the indicated single gRNA, followed by Tyr expression evaluation at 48 hpi. (A) Representative images of Tyr production in embryos injected with dCas9-KM systems. Scale bar=2.5 mm. (B) The qPCR validation of Tyr expression in embryos co-injected with dCas9-KM systems. Data are presented as mean ± SEM (*n*=4 per group), ***p*<0.01 versus gNC group (one-way ANOVA test). (C) Embryos with different phenotypes were counted and compared with the total developed ones after injection. Total embryos evaluated for each group (*n*) is shown above each column. Ns, no significant differences versus control.


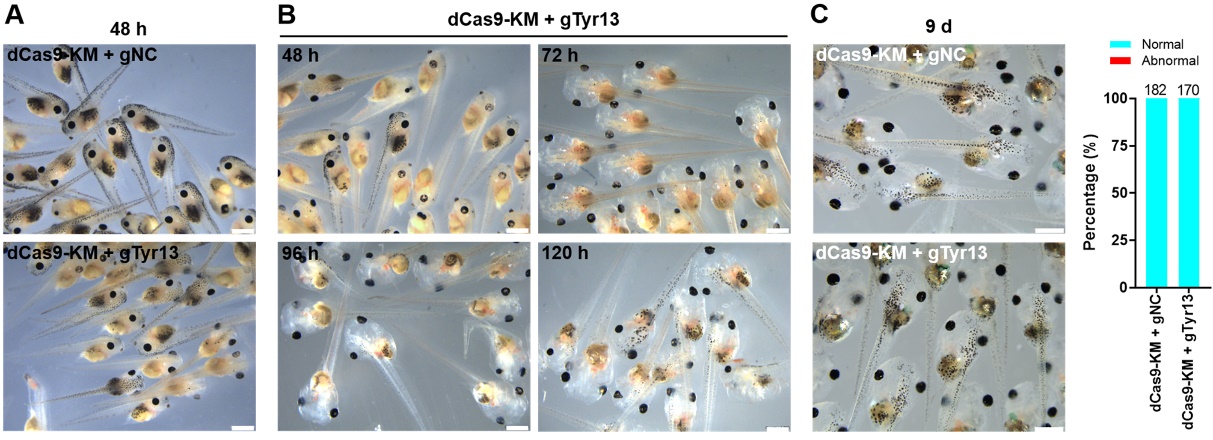


**Figure S10. Effective duration of dCas9-KM-induced endogenous gene knockdown in embryos of *X. tropicalis*.** The dCas9-KM mRNA was co-injected into the animal pole of *X. tropicalis* fertilized eggs at one-cell stage with gNC or gTyr13, followed by Tyr expression evaluation at different time points. (A) Representative images of Tyr production in embryos co-injected with dCas9-KM systems at 48 hpi. (B) Embryos with albinism phenotypes isolated from gTyr13 group (A) were further raised for an extended observation till 120 hpi. (C) Representative images (left) and quantification (right) of tadpoles with or without pigmentation loss at 9 days post-injection. Scale bar=1 mm (A and B) and 750 µm (C, left).


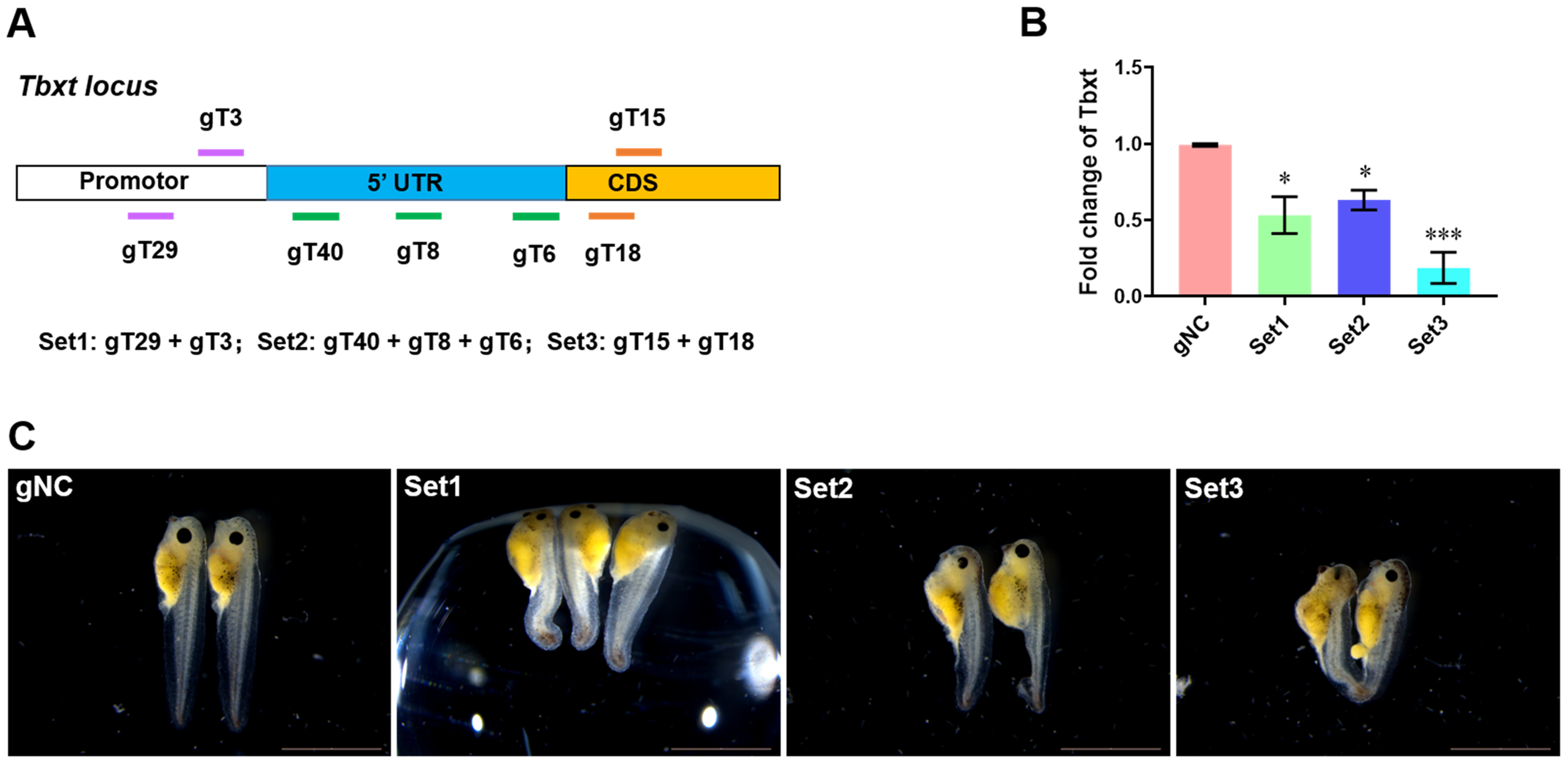


**Figure S11. Effects of dCas9-KM system on tbxt expression in embryos of *X. tropicalis*.** (A) Schematic illustration of dCas9-KM system-related gRNAs targeting the DNA locus of endogenous tbxt gene. Fertilized eggs were co-injected with dCas9-KM mRNAs and indicated gRNA sets, followed by embryo evaluation at 48 hpi as follows. (B) The qPCR validation of tbxt expression in the dCas9-KM-injected embryos with different gRNA sets. Data are presented as mean ± SEM (*n*=3 per group), **p*<0.05, ****p*<0.001 versus gNC group (one-way ANOVA test). (C) Representative images with the abnormal tails in embryos injected with tbxt targeting gRNA sets compared with gNC. Scale bar=2 mm.


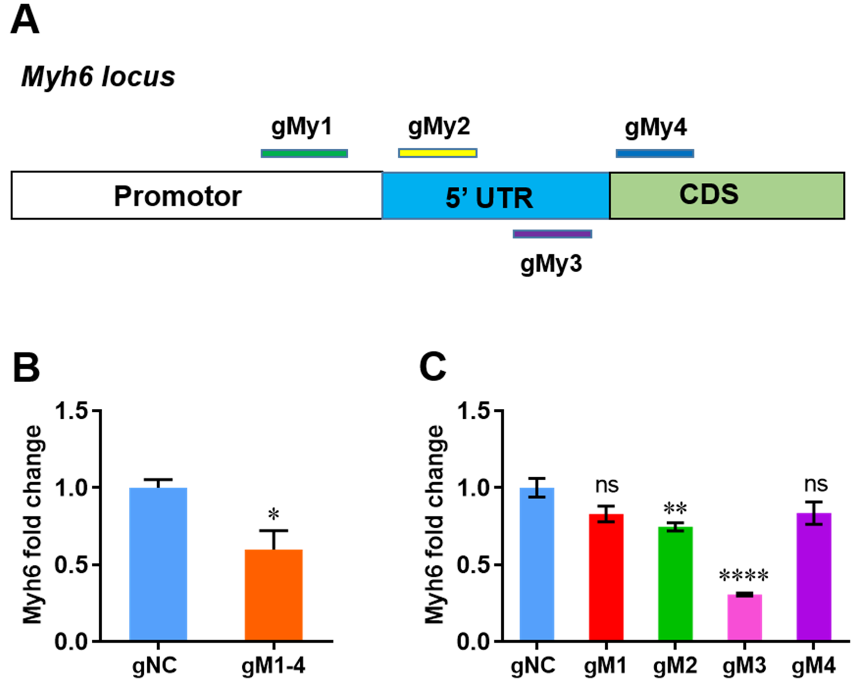


**Figure S12. Effects of dCas9-KM system on the expression of Myh6 in embryos of *X. tropicalis*.** (A) Schematic illustration of dCas9-KM system-related gRNAs targeting the DNA locus of endogenous myh6 gene. Fertilized eggs were co-injected with dCas9-KM mRNAs and indicated gRNAs, followed by embryo evaluation at 48 hpi as follows. (B and C) The qPCR validation of myh6 expression in the dCas9-KM-injected embryos with gRNA sets (B) and single gRNA (C). Data are presented as mean ± SEM (*n*=4 per group). **p*<0.05 versus gNC (Student’s t test for B). ***p*<0.01, *****p*<0.0001 versus gNC group (one-way ANOVA test for C). Ns, no significant differences versus control.


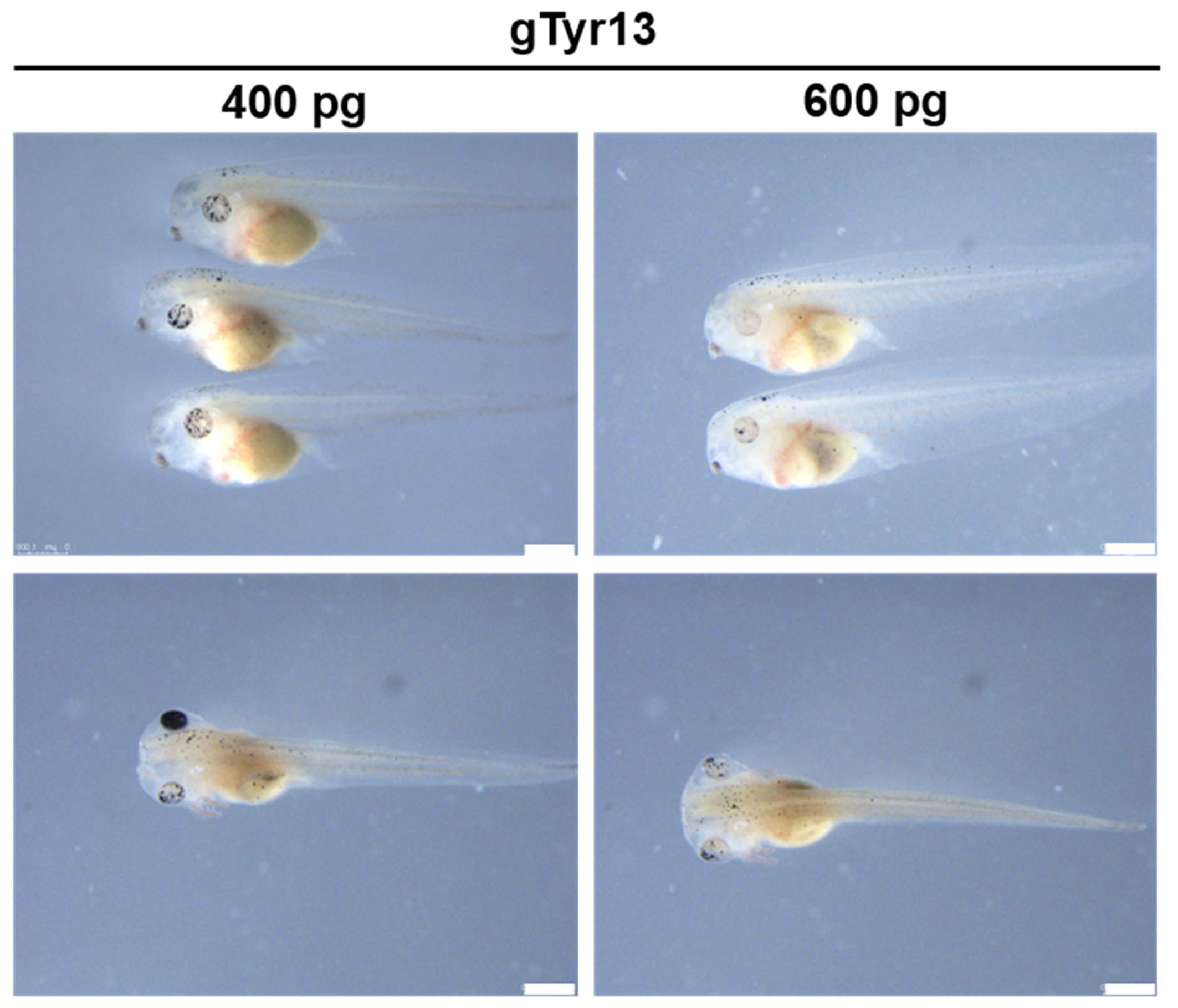


**Figure S13. High dose of dCas9-KM system induces a complete ablation of pigmentation in *X. tropicalis* embryos.** Fertilized eggs were co-injected with dCas9-KM mRNAs (300 pg/embryo) and gTry13 with high dose (400-600 pg/embryo), followed by picture capture at 48 hpi. The most representative images of severe albinism tadpoles with complete ablation of pigmentation were isolated. Upper panel, dorsal view. Lower panel, lateral view. Scale bar=1 mm.


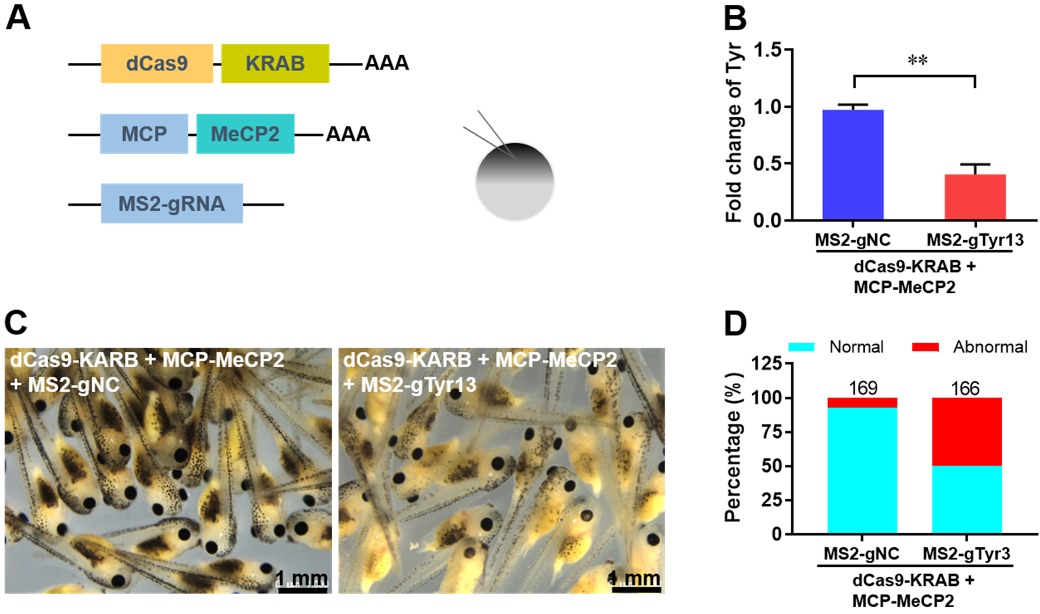


**Figure S14. Effects of MS2-MCP system on dCas9-KM-induced tyr knockdown in embryos of *X. tropicalis*.** (A) Schematic illustration of the experimental setup used to analyze the effect of MS2-MCP system on dCas9-KM system in *X. tropicalis* embryos. The MS2-gTry13 were injected into the fertilized eggs together with dCas9-K and MCP-MeCP2 mRNAs, followed by embryo evaluation at 48 hpi as follows. (B) The qPCR validation of Tyr expression in the injected embryos. Data are presented as mean ± SEM (*n*=5 per group). ***p*<0.01 versus control group (Student’s t test). (C) Representative images of tyrosinase expression in the co-injected embryos at 48 hpi. Scale bar=1 mm. (D) Embryos with different phenotypes were counted and compared with the total developed ones after injection. Total embryos evaluated for each group (*n*) is shown above each column.
